# Supplementary material for: The origin of the odorant receptor gene family in insects
Source: eLife. 2018 Jul 31;7:e38340. doi: 10.7554/eLife.38340 (PMC6080948; doi:10.7554/eLife.38340)
Supplement: Supplementary file 1. — Table S1 Transposable element repeat class analysis of the Thermobia domestica genome assembly. Table S2 Details of the Thermobia domestica OR family genes and proteins. Columns are: Gene – the gene and protein name we are assigning (suffixes, which are not part of the name but indicate features of the gene model, are C – C-terminus missing, F – assembly was repaired, J – gene model spans scaffolds, * - one or more join across scaffolds made on the basis of comparison with an ortholog in Ctenolepisma longicaudata or a close intact relative in Thermobia); Scaffold – the v1 genome assembly scaffold ID; Coordinates – the nucleotide range from the first position of the start codon to the last position of the stop codon in the contig/scaffold; Strand –+ is forward and - is reverse; RNA – number of independent pairs of reads from Missbach et al. (2014) and 1Kite (Misof et al., 2014); Introns – phases of introns (bold indicates those supported by Missbach et al. cDNAs, or their raw RNAseq reads, or those from 1Kite); % - percent identity for most apparent 1–1 orthologs with Ctenolepisma longicaudata; AAs – number of encoded amino acids in the protein; Comments – comments on the gene model. Note that Orco is Orco2, Or1 is Orco1, and Or9 is Orco3 of Missbach et al. (2014). Table S3 Details of the MhraOr family genes and proteins. Columns are: Gene – the gene and protein name we are assigning (suffixes, which are not part of the name but indicate features of the gene model, are F – assembly was repaired, J – gene model spans scaffolds, * - one or more joins across scaffolds is based only on sequence similarity to the other proteins); Scaffold – the v1 genome assembly scaffold ID from i5k; Coordinates – the nucleotide range from the first position of the start codon to the last position of the stop codon; Strand –+is forward and - is reverse; RNA – number of independent pairs of reads from 1Kite and the i5k pilot project (single reads from Missbach et al. (2014) for the relate [file elife-38340-supp1.docx]

**Supplementary File 1**

Table S1 Transposable element repeat class analysis of the *Thermobia domestica* genome assembly

| **Repeat element family** | **Total number elements per family in genome** | **cumulative length [bp]** | **% of genome assembly** | **% of cumulative element length** | **% of element number** |
| --- | --- | --- | --- | --- | --- |
| **Class I - Retrotransposons** | **2,189,237** | **611,713,341** | **10.97** | **20.7** | **21.9** |
| SINEs | 1,216,723 | 206,323,846 | 3.7 | 7.0 | 12.2 |
| ALUs | 0 | 0 | 0 | 0.0 | 0.0 |
| MIRs | 0 | 0 | 0 | 0.0 | 0.0 |
| LINEs | 560,608 | 289,478,914 | 5.19 | 9.8 | 5.6 |
| LINE1 | 0 | 0 | 0 | 0.0 | 0.0 |
| LINE2 | 33,935 | 15,264,789 | 0.27 | 0.5 | 0.3 |
| L3/CR1 | 50,233 | 24,178,122 | 0.43 | 0.8 | 0.5 |
| LTRs | 411,906 | 115,910,581 | 2.08 | 3.9 | 4.1 |
| ERVL | 0 | 0 | 0 | 0.0 | 0.0 |
| ERVL-MaLRs | 0 | 0 | 0 | 0.0 | 0.0 |
| ERV_classI | 0 | 0 | 0 | 0.0 | 0.0 |
| ERV_classII | 0 | 0 | 0 | 0.0 | 0.0 |
|  |  |  |  |  |  |
| **Class II - DNA Transposons** | **2,140,882** | **813,267,020** | **14.58** | **27.6** | **21.4** |
| hAT-Charlie | 97,473 | 25,645,988 | 0.46 | 0.9 | 1.0 |
| TcMar-Tigger | 30,684 | 9,434,429 | 0.17 | 0.3 | 0.3 |
| **Total Classified Transposons** | **4,330,119** | **1,424,980,361** | **25.55** | **48.3** | **43.4** |
| **Unclassified** | **5,656,688** | **1,524,050,197** | **27.32** | **51.7** | **56.6** |
| Total Repeat Families | 9,986,807 | 2,949,030,558 | 52.87 | 100 | 100 |

Table S2 Details of the *Thermobia domestica* OR family genes and proteins. Columns are: Gene – the gene and protein name we are assigning (suffixes, which are not part of the name but indicate features of the gene model, are C – C-terminus missing, F – assembly was repaired, J – gene model spans scaffolds, * - one or more join across scaffolds made on the basis of comparison with an ortholog in *Ctenolepisma longicaudata* or a close intact relative in *Thermobia*); Scaffold – the v1 genome assembly scaffold ID; Coordinates – the nucleotide range from the first position of the start codon to the last position of the stop codon in the contig/scaffold; Strand – + is forward and - is reverse; RNA – number of independent pairs of reads from Missbach et al. (2014) and 1Kite (Misof et al. 2014); Introns – phases of introns (bold indicates those supported by Missbach et al. cDNAs, or their raw RNAseq reads, or those from 1Kite); % - percent identity for most apparent 1-1 orthologs with *Ctenolepisma longicaudata*; AAs – number of encoded amino acids in the protein; Comments – comments on the gene model. Note that Orco is Orco2, Or1 is Orco1, and Or9 is Orco3 of Missbach et al. (2014).

| **Gene** | **Scaffold** | **Coordinates** | **Strand** | **RNA** | **Introns** | **AAs** | **%** | **Comments** |
| --- | --- | --- | --- | --- | --- | --- | --- | --- |
| OrcoFJ | 306089 | 32493->32712 | + | 7 | **0-2-0-0-0** | 484 | 88 | Join across 7 scaffolds |
|  | 252574 | <1->6187 | + |  |  |  |  | Exon1 split across scaffolds |
|  | 166573 | <1->9235 | + |  |  |  |  | Assembly repaired |
|  | 470722 | <1->6248 | + |  |  |  |  |  |
|  | 543310 | <1->8541 | - |  |  |  |  |  |
|  | 55767 | <1->9362 | + |  |  |  |  |  |
|  | 116122 | <1-15328 | + |  |  |  |  |  |
| Or1J | 230086 | 11865->19068 | + | 6 | **0-2-0-0-0** | 478 | 82 | Join across 4 scaffolds |
|  | 33241 | <1->4012 | + |  |  |  |  |  |
|  | 277514 | <1->12364 | - |  |  |  |  |  |
|  | 78038 | 47065->47792 | - |  |  |  |  |  |
| Or2 | 305721 | 36346-52500 | + | 0 | 0-2-0-0-0 | 471 | 53 | Within one scaffold |
| Or3 | 147262 | 7163-30763 | - | 3 | 0-**2**-0-0-0 | 470 | 50 | Within one scaffold |
| Or4 | 251839 | 15006-35080 | + | 4 | **0**-2-**0**-**0**-0 | 452 | 77 | Within one scaffold |
| Or5J* | 306089 | <1-13109 | - | 5 | **0**-2-**0**-**0**-0 | 454 | - | Join across 2 scaffolds |
|  | 395000 | 11040->32100 | - |  |  |  |  |  |
| Or6J* | 287065 | 48->7995 | + | 4 | 0-2-0-**0**-0 | 462 | - | Join across 3 scaffolds |
|  | 315037 | <1->10435 | - |  |  |  |  |  |
|  | 459954 | <1-7255 | + |  |  |  |  |  |
| Or7JC* | 27200 | 7160->15500 | + | 3 | 0-2-0-**0**-0 | 455 | - | Join across 3 scaffolds |
|  | 86519 | <1->11660 | + |  |  |  |  | Final exon unidentified |
|  | 127360 | <1->11085 | - |  |  |  |  |  |
| Or8J* | 453422 | 7683->20014 | + | 2 | 0-**2**-0-0-**0** | 462 | - | Join across 2 scaffolds |
|  | 11168 | <1-3494 | + |  |  |  |  |  |
| Or9J | 580933 | 6878->16065 | + | 4 | **2-0-0-0** | 485 | 72 | Join across 3 scaffolds |
|  | 571356 | <1->10291 | + |  |  |  |  |  |
|  | 308436 | 20515->38961 | - |  |  |  |  |  |
| Or10J* | 522795 | 673->1573 | + | 2 | 2-0-0-**0** | 456 | 68 | Join across 4 scaffolds |
|  | 121300 | <1->8137 | - |  |  |  |  | Exon1 split across scaffolds |
|  | 249181 | <1->3864 | + |  |  |  |  |  |
|  | 253685 | 6187->11780 | - |  |  |  |  |  |
| Or11J* | 544642 | <1-24846 | - | 3 | 2-0-0-**0** | 468 | 67 | Join across 2 scaffolds |
|  | 606400 | 15713->21228 | - |  |  |  |  |  |
| Or12J* | 455811 | 8829->21699 | + | 3 | 2-0-0-0 | 446 | 67 | Join across 2 scaffolds |
|  | 80727 | 2968->18501 | - |  |  |  |  |  |
| Or13J* | 432607 | <1-6113 | - | 0 | 2-0-0-0 | 474 | - | Join across 2 scaffolds |
|  | 396309 | <1-1631 | + |  |  |  |  |  |
| Or14J* | 432607 | 15872->19919 | + | 2 | 2-0-0-0 | 461 | - | Join across 4 scaffolds |
|  | 451874 | <1-6796> | + |  |  |  |  |  |
|  | 122756 | <1-4034> | - |  |  |  |  |  |
|  | 150848 | 955->7401 | - |  |  |  |  |  |
| Or15aJ | 76671 | 1168->38506 | + | 0 | 2-**0**-**0**-**0** | 470 | - | Alternatively spliced |
| Or15bJ | 76671 | 10470->38506 | + | 0 | 2-**0**-**0**-**0** | 470 | - | Alternatively spliced |
| Or15cJP | 76671 | 24385->38506 | + | 1 | 2-**0**-**0**-**0** | 456 | - | Pseudogenic first exon |
|  | 615227 | <1->6840 | + | 1 |  |  |  | Join across 4 scaffolds |
|  | 72518 | <1->5809 | + | 1 |  |  |  |  |
|  | 489799 | <1-4081 |  |  |  |  |  |  |
| Or16JC* | 229400 | <1-6927 | - | 0 | 2-0-0-0 | 448 | 63 | Join across 3 scaffold |
|  | 238675 | <1->4045 | - |  |  |  |  | Final exon unidentified |
|  | 52482 | <1->12620 | - |  |  |  |  |  |
| Or17J* | 162524 | <1-14232 | - | 0 | 2-0-0-0 | 476 | 62 | Join across 4 scaffolds |
|  | 154846 | <1->4087 | - |  |  |  |  |  |
|  | 52482 | <1->12620 | - |  |  |  |  |  |
|  | 366562 | <1-8024 | + |  |  |  |  |  |
| Or18 | 369759 | 3969-17958 | + | 2 | 2-0-0-**0** | 490 | 50 | Within one scaffold |
| Or19 | 192862 | 1877-45187 | + | 2 | **2**-0-0-0 | 464 | - | Within one scaffold |
| Or20J* | 616889 | <1-13166 | - | 1 | 2-0-**0**-0 | 464 | - | Join across 2 scaffolds |
|  | 47847 | <1-4266 | + |  |  |  |  |  |
| Or21 | 317857 | 40091-63654 | - | 2 | 2-**0**-0-0 | 464 | - | Within one scaffold |
| Or22J* | 544642 | 32803->39175 | + | 0 | 2-0-0-0 | 468 | - | Join across 2 scaffolds |
|  | 231032 | <1-19933 | + |  |  |  |  |  |
| Or23 | 47847 | 7399-41943 | + | 1 | 2-**0**-**0**-0 | 473 | - | Within one scaffold |
| Or24a | 317857 | 67501-101689 | - | 0 | 2-0-0-0 | 468 | - | Alternatively spliced |
| Or24bP | 317857 | 67501-96047 | - | 0 | 2-0-0-0 | 470 | - | Alt. spliced, but pseudogenic |
| Or25FJ* | 407319 | 1414->1905 | + | 4 | 2-**0**-**0**-0 | 461 | 66 | Join across 3 scaffolds |
|  | 6820 | <1->16036 | + |  |  |  |  | Assembly repaired |
|  | 253685 | <1-4222 | + |  |  |  |  |  |
| Or26 | 214043 | 13203-26863 | - | 2 | 2-0-**0**-0 | 454 | 66 | Within one scaffold |
| Or27aJ* | 377263 | 14874->20009 | + | 2 | **2**-0-0-0 | 438 | - | Alternatively spliced |
| Or27bJ* | 125151 | 11497->37484 | + | 0 | 2-0-0-0 | 438 | - | Alternatively spliced |
| Or27cJ* | 125121 | 19894->37484 | + | 0 | 2-0-0-0 | 438 | - | Alternatively spliced |
| Or27dJ* | 63428 | 4673->17184 | + | 0 | 2-0-0-0 | 440 | - | Join across 2 scaffolds |
|  | 599414 | 6753->10940 | - |  |  |  |  |  |
| Or28J* | 139075 | <1-21269 | - | 1 | 2-0-0-0 | 508 | 68 | Join across 5 scaffolds |
|  | 282012 | <1->24015 | - |  |  |  |  |  |
|  | 494962 | <1->34250 | - |  |  |  |  |  |
|  | 354737 | <1->12842 | + |  |  |  |  |  |
|  | 484088 | 13261-20877 | - |  |  |  |  |  |
| Or29J* | 231873 | <1-5342 | - | 0 | 2-**0**-**0**-**0** | 463 | 56 | Join across 2 scaffolds |
|  | 211379 | <1-20094 | + |  |  |  |  |  |
| Or30FJ* | 2208 | <1->18453 | - | 3 | 2-**0**-0-0 | 445 | 54 | Assembly repaired |
|  | 470596 | <1-1699 | + |  |  |  |  | Join across 2 scaffolds |
| Or31aJ* | 8260 | 1152->3911 | + | 0 | 2-0-0-0 | 464 | - | Alternatively spliced |
| Or31bJ* | 293478 | 5159->11177 | + | 0 | 2-0-0-0 | 448 | - | Alternatively spliced |
| Or31cJ* | 293478 | 10589->11177 | + | 0 | 2-0-0-0 | 455 | - | Alternatively spliced |
|  | 474626 | <1->27135 | + | 0 | 2-0-0-0 |  |  | Merge across 2 scaffolds |
| Or31dJ* | 474626 | 4472->27135 | + | 0 | 2-0-0-0 | 460 | - | Alternatively spliced |
| Or31eJ* | 474626 | 8819->27135 | + | 0 | 2-0-0-0 | 449 | - | Alternatively spliced |
| Or31fJ* | 474626 | 14213->27135 | + | 1 | 2-0-0-0 | 465 | - | Alternatively spliced |
|  | 57694 | <1->5992 | - |  |  |  |  | Joined across 3 scaffolds |
|  | 448088 | 13365->20877 | - |  |  |  |  |  |

Table S3 Details of the MhraOr family genes and proteins. Columns are: Gene – the gene and protein name we are assigning (suffixes, which are not part of the name but indicate features of the gene model, are F – assembly was repaired, J – gene model spans scaffolds, * - one or more joins across scaffolds is based only on sequence similarity to the other proteins); Scaffold – the v1 genome assembly scaffold ID from i5k; Coordinates – the nucleotide range from the first position of the start codon to the last position of the stop codon; Strand – + is forward and - is reverse; RNA – number of independent pairs of reads from 1Kite and the i5k pilot project (single reads from Missbach et al. (2014) for the related species *Lepismachilis y-signata* are shown in parentheses); Introns – phases of introns (bold indicates those supported by RNAseq reads); AAs – number of encoded amino acids in the protein; Comments – comments on the gene model.

| **Gene** | **Scaffold** | **Coordinates** | **Strand** | **RNA** | **Introns** | **AAs** | **Comments** |
| --- | --- | --- | --- | --- | --- | --- | --- |
| Or1F | 8919 | 2606-19798 | + | 13 (5) | **2**-0-**0**-**0** | 491 | Exon4 from raw reads |
| Or2FJ | 72278 | <1-1364 | - | 7 (0) | **2**-**0**-0-0 | 498 | Repair ambiguous bases |
|  | 8919 | 25482->37666 | - |  |  |  | Join across 2 scaffolds |
| Or3F | 22483 | <6015->13286 | + | 10 (0) | **2**-**0**-**0**-**0** | 488 | Multiple assembly repairs |
| Or4FJ* | 50778 | 1413->14342 | + | 4 (0) | 2-**0**-**0**-0 | 469 | Multiple assembly repairs |
|  | 22483 | <28546->35391 | - |  |  |  | First two scaffolds overlap |
|  | 151641 | 210->1386 | - |  |  |  | Join across 3 scaffolds |
| Or5FJ* | 200277 | <1->737 | - | 4 (0) | 2-0-0-0 | 474 | Multiple assembly repairs |
|  | 47247 | <1->18550 | + |  |  |  | Join across 3 scaffolds |
|  | 27669 | <1-24594 | + |  |  |  |  |

**FASTA format proteins for the newly described ORs:** Suffixes, which are not part of the gene/protein name but indicate features of the gene model, are C – C-terminus missing, F – assembly was repaired, I – internal regions missing, J – gene model spans scaffolds, P – pseudogene. All proteins of the newly described ORs and the alignment used to reconstruct the gene tree are available on Dryad.

**4 *Ladona fulva* ORs.**

>LfulOrco

MDKADRRSKRKVFPHAWDKSPKREKKSSLATDLSIHIRMMRLAGHFLPDFNPDSDSKFSLVWAIYSFTHMALMSLQFIAMAVNMVKHFDEVSALTTNAISLLFYLHGPIKVFYFAIYRRRFYKTIRSWDSSLSDQGEGAAMNKASNGIESIDHSITECMKKAQFGAQEWAFENSNVRFRKLAVRRMRKLLVSVMSGSAAAAAFWSLRPFLGLGHYSRKLVMNEGNASLPMDGGQWYLLVDATYPWETSSNVAYALTFLYQVYWIVFCLAQINLLDLLLCSWLIFACEQILHLKDILKPLMQKSQDKNLQSADAVLFKRKGALFQNELSLKRGSTLERTSTLSILQVNPTAHNGYSALSDDESFGLNKGPEDGYFHMKDMSGRNGSNQSLEMRMYQNNNQEISVHCCIKYWVEKHKHIVRFVDCIEDSYGMALLIHMLTSTITLTLLSYEATNISSVDMHALTVITYLLYTLGQVFLFCIYGNKLIEESTSVMQAAYESPWYDCTEEAKAFIQIVCQKSQRAMSISGAKFFTVSLDLFASVLGAVVTYFMVLIQLK

>LfulOr1

MARRRIEYLRFHRDLLRFMWLFPSTQKKSWKTTVAIVSHFSMMAALLFQLAGELMDFVIHSHEIDIWTDVACMAVFSCSSIVRLVILFYKKDDLYNLMSKWEDQFVRKSPFNPARKDVERLIFKTKAYILTTTIITMLIAFHWCVYPIAVAILFPEQSRKLPLRAWYPFDMYASPIYEFIYLMQIIRGVFGTYIAVYWETTLMSFCILTYYEAKKLKEHLRSIATDFKEGEEDKIEDYLTKCILHHRTTVRFVKRIGETYSHALVVELFYLVVPMCFTALKVSSFFGNDTTKYLNWLEFFLAGLIQLFILCLGGHIVTSQLISIQGAAYEACARVSGRIRWKLAFMMLHSNLEMKITGGNFFILSMKTFKTIVGMSFSNFIVLRQLKESKYSTGLQS

>LfulOr2

MGRQKIRYLTVLGNIFNVMCLLPMDNRRLKIRSLVLLYHIAILVLLLLRLAGEFMDFVQHINHTYDWPRIASIMILTIMSILRIFLYAQRKEKNLRILQLWEKLFTKNLKLGTRIEDIQKDIRTCNMYSLAMIVATMGGTMHWALYPLVLGYFTPGKRVLPLRTWYPIDLYSSPTYEIVYAFQFIGTLLTTYNPALSATSFLTFSILVQKQVQELKKQLKAIGVTGKHIGASVEPLGERRQMIEADVLRCIKFHSGIIRFVREMQNYYSSVLVIEYLSFTLPLCFTIIEATSGSLHDSIAWAEFFVTCSLTILLYCWNGSRLTLQLLSIQDAISEALCGHLTNEMRWNLRFMMHQSNVEFTVTGGRLFITSMETFKNLIGFAVSNYLFFKELKG

>LfulOr3

MKKIILFFRLSGHWFPFQILEKCTSSKNGLVFCLIDLFNKLGHPQAAIKNVITTFYAIQTLGRVIFVAVRGTYIWQLLMSFGREITEINAKDKQRTTKKSFILMQNGENNDYFLYIFLMIVLGANLTGIIFTITPFVISYSHAYYQSTNSLPNETVIQRPLVFDAWYPFDMYNSPSYEMVLAIQALCGFVWINMMSSSDAIFLSILAKVLAEIRLLNLRTNKIQLLSNFRDIETRNKLFMKLLKDWVLHHQRIMKMCREIESTFSGFILMTFLFNGGSLSLLAYNVARFQDTISIISVSGYFCVVSFQLLVMSHYGQKVTDKSLNIKKNIYSIPWWKFSKYVRSTFLIILENTEQPITFTGMGYFNLSMEFLLSVFQAAFSYFIILIQLT

**47 *Ephemera danica* ORs**

>EdanOrcoF

MIQMIFTPLTNRITMMQMSHGGKGAHRGLAHDLRTLLMLMQLAGHFMPDYSVAKQQQQQSGRGLLRYVYSLVNIGLETLHFLCLLAQLLIQFGDTAALVSNTLTMLFFLHGVTKVTYFALKRKQFYRTMLVWESSHSHPLFVESDVKHRALGEREMLRLMRYVLVATGVTALLWAAHPFLSTDATPPSPDNATLLSTNVTVLGVDLLSSTAVTPVVLQLQGPKLMVNAWYPWDPYSSSFLYVITYLFQLYWLVFCICQVNLMDTLFCSWLIYGCEQLRHLKEGMQGLMQLSASEAALATAGDVFPDTREASEMADNASNLFLRSTSRVFPTMTGTARVRHTVTPEMLGFRNLEPPLPGQNAEQQQELLRQDMVVRSAIKYWVERHKHILRFVESIGETYGMALLLHMLTSTVTLTLLSYEATKIAGVNLYAFTVIGYLCYTLGQVFLFCVFGNRLIEESVSVMHAAYSCPWYDGSEEAKTFVQIVCQQCQRPMSVSGAKFFTVSLDLFASVLGAVVTYFMVLIQLN

>EdanOr1

MFSSYRMSSLSSYSQLYLKVLRVSGVWPWKTQHEPWQRILAHAYTTYAALAVTGSFLGTSLVFVMMHLSEWQAVLGDLWLKVSLASISLKLVLFLMHRKEINTLLNSSPKFISTSVPSHLNNYKTEAIQRSALQIALMIIIAIVAYFYFQADIFIVDIAHLLKPTKNTQGNTLYILGTLLLLAVERCALVILMLFHLGAFGFYLTYLRFIEGYLDHLKDSIIKISVEPKLQKDTLFTNYNASTTDQEVRYCVSLHQFIIGLVDRGNAAFSTQMLFKLWRSLVVLCLTAKELTNENQPFGNKLTITLIFLANIFELFNVCWLADVIQSKMRHAAVRDDMVTAQNGSEGFDEDDNWITMQAMSYRCQMPLDLRAGPYYHLSIHTFSAILGLSFTYFIFLTSVKT

>EdanOr2

MSTIYRTYYIKKKTNSIRNVSSKEKMNFIYNININDVFFKPIYFLNFIGFWNDEKSNFQKITSNLFTFVCILSTVAHSIFNFIDDEKTWLLLGFKLVNAFHHAIIAIVKFHLVNIKKESIFQLLANCLHNNISAPKILVTQIYHFDNRFKKYNKALVKIVTILVLLILYTPIYIFIYMYTEVKWDVKSILSFITQGLGTVFLINVTLSYSLIYITLMLTLCKILDHMNDVILTLRNATHRELSDCNENISKKLKYIVQTHQRAIRFADGINEIFSYNALLDMCLALALVIVSVIDFKSSNLFPYLNVHAFYVLMDFCTSVLLAIYCYFGSMVIEKSGKKIAENFNSYQRQNWNSDNQQITKIILIRVRFPIRILAGQIFTISNKTLAMLAGYAASYTIIFFQIQIKHKNTDTTIEQNSTVDAIVFK

>EdanOr3C

MMIDSPTTQRTTNYNLDIDMALAWPARILQIFGLWTENSNSRSRLICSRLLLACAMISSVITTVELIRTVQYHLLDITFVFTTTLRYLAWFYQIIIFATKRQYITKLLQECYYNIPAPIYLNRNIKKLKHNQSRILNRPLAMILITLIIITSFITSYDGDGYFSLTLLKIVVFRVLYASMHLTKLGFNLMYLAWLQTTLNVLDHINDVICTLHCKRKSLGPRKMDEKEQHQIAQTLQYAANLHQRVAKFINGFNRIYSSIMFLYIWTTHLMFAVIVLKIAKQEVLYSLTGMFVIIVSTAVSYLHTFLICLKATCIAEQSGTKIRNNLSHVAWWGWSRINLNTRNVIVTMTKHEMQIRAGSIFICSLSNFIT

>EdanOr4

MNIVLFLQQSALIWRQPMMKFSLDIDYILKWPKFLLQIFGLWNDNSSKICLFLLRSMLFFLVISTILSSFNEIFTTDHQLLMPANFVAQTIRYLSWLQKIFIYLTKRKKMSDLLASSFCNITAPTFLRSYTKQFRLHSNKIMNKSYIMRYVYIILFILIIIFLEHFDLSSLAMIVIFIISRLLFALLHIQALGYSIMYLSWLELSLCQLEHMNQVLRTLQYSRRNVLKKQKLQNSRNQIRITETLRYVIQLHQRVARFINGLNHIFSFVMLMELWSTHVIFSVTFVKLVFEGLLHDMALTIAIVISTILIYFYMGLICWKATCIVEQSSSKLQNNLCNVPWWEWNSSNLNLRNCMFSISMREMQIRAGPIYNFSLPTFATILSVSLYYFIFLYQLGDGFK

>EdanOr5

MKFSIDFERILMWPQIPLKIFGLWNANSSKFTLFLLRTALTFIVLSIILNTIVLIINADKNLISISIVANGIRFVAWIQRIIFFLTKRQAMVDLLSQYFHDISAPANFRRQIDRFRLKTSKLMSKTYVMRYFFAILLIVSITLYEELDVSSIPMIFIYFIVRLLFILLHVQILGFNLMYITWLQLTLCLLEHMNEIVRTLHCTRNGLGPQKWNSAHQARIKKTLRYTIQLHQRAAKFVCGLNQMFSNVMMMDLWSTHVVFSIAFVQIAIEGMLHNIATSLATVMSTLLIYFYMALICQTSTDIVEQSGRKLQDNLCHVPWWGWNQSNLNIRNGIYSSSMHGLQIKAGPIYNFSLATFATVLSVSFYYFMILYELNTTRI

>EdanOr6P

YNVNVIQSLLWPRRILKFFGLWSEDAIRYRILRQWSLLFVTFITLTWISATQIVHSQSSLRELTEMAVLITSHVTWVLKMHVLLTKGRDLTDLLHSCFRNIQAPSHLKRQVWNFX MKHPGKFIAFLVIMSTLLSLPISPIRELNTGFMLLNVKFFLMILAQSVFIITRVQYYTFNVLYVSLLIMVTKTLNHLNEVLSLLHDDDHDLQDRAQSNKDYQQLTKRTLNYVAQTHQRISRLTEGLNKILSNVIFMDVVSTHLVTCRLILEFFKKDELEVRNVVFFVAICILMNSYLFSICWLATDIKEKSGAKLQNNLWHSTWWEWESSNVRLSNAVVARAKRDLLMSAGPFYSLSVEFFSGMLGVSFSYSIILYQLQDYVQ

>EdanOr7

MVFLWLDNPTRLQNTCSKICHILVAFSFILTVVFVCIVSVTIVFSFIRSLTLTLKMFHTAKILNFVFFKQKCVIDSLKNSLSSISAPIFLSKHVYDFIDSIENVNKKWKMFYKSFSISLNFAAAIFYLFRVYMADFEFFHVLLLFFLGIAANFILFMHLGLHILQITWIRIICHVLDHINNVILTLHHEERKLNNETTNLVYQNRVARTLDHIGQIHQTALKLVNGFNTYFDTLLFVNIMTDIASVVLTPMQQIMRDEKQSLIRDYSVPVSLSLSFALLLIKSWFGSLIIRTGSTVALHSMSNARWQSWNIENKVEADKVVAMFQSELRLTAGPFYYLSMKTFSSVVGAAFSYTFLIKPLYSMMTPNN

>EdanOr8I

MFNMVFFWLDNPSRIQNICSKFCHILAAFSFIVTVVFFITSVTTVFSFARSLAITLKMFYAAKILNIVFFKQKCVIESLNNSFSDISAPIFLSKHVYDFIDSIENVNKKWKMFYKSFSISLNFAAAIFYLFRVYMADFEFFHVLLLFFLGIAANFILFMHLGLHILQITWIRIICKVLDHMNNVILTLHHEERKLNNETTNLVYQNRVARTLDHIGQIHQTALKLVNGFNTYFDTLLFVNIITDIVYVVLTPMLQIIQEEKQSLTRDYFVLISLSLSFASLLIKSWFGSLIIRTVVGVAFSYTLLIKSLYDDTE

>EdanOr9

MKRLKFIRIICPRLAIILLVLFVFLEMVDLVTSDQSLLHISYTIMLIPRQLCTIRKIYIFIFSSEFRRFIYDSEFLRFSGPRHLSPLIQKFQTRCYETAKLSIVLIITFLIGFIPIWNLSFFNWEGTTFINITDNAVVKPKLQFSIWHPFDFNQNKLIFNVILVLQSLIMIFAGVLITSFNIFYTSLAAMCSRLFDHLYDVLLHNQLLDNTFYNLRRCQNLNDLNISQSDIFLSHEPISFPKKEIKDESGISALNEGLHDWNSKHKYLESDDKVMETLRYAVQHHQRSVRFVHGVNKIFSGVMLLDIWTVVLIMCVLAFQVVSSEEGALRTSALAKNFILHLIFFALICWASNEVMVKSSAPIHAAANHACWLCWRTSTRNVVHCLATYSYFPLRLSAGPFYTLSLETFTALLSLTFSYFMFLYQIYESKDKT

>EdanOr10

MNWPRRFMQCINMWYHDAFPGSKKHLHRFLRFLQKLILKITYFSIVTLWLANCSYLLITDRHQPFVKNTHSFLIIIRQFFGFCKTVVVHRNKRQMERLLKESFCDITVPTCLGRQLNKYRKSFKKSHKILLINTVYIIITMIPIVLMSPLALTKFLKIENFDIKLLDWNVSTIFPLLIYVIQIFAIVPIVIMMMGSSLYYLGLLCLANGMLDHLNLVLETLHEKRSKFKRRGMTHTNNPEDSPGYQQRVGATLKYIVQHQIKVTRYLKGINNIFSNVLLFEVLSLHVVFSVSAFAIVTAGITSSLVIGMIPALLNYFFNFAFCCWAANDVNVKCSDTLHKSTTHMASWTWDRDNKLLGQCITEYSKVVLRVSAGPFYSLSLNTINTVLSASFSYLLLLYNLNGTL

>EdanOr11

MDLWPENIFPGFKRTLFDRCVWIFQKIMFVFSVTLLSFLCISNITHLLLVDRGKTFLNFTQTLMIICRQTFGLFKMYTYFKNKRELRHLLNDTFYDVPAPTCLRQQIKKYKQSCLKTPKVVSISSIIIIVALIPVLIGAPLQVTPVLKYQNSELVLLNLNVPKIIPQLIYIAQVLGIILVFNVAFGCIIIYIALLTIAHRMLDHLHEVLKTLHHGHKKIGKNKMLHDHQQRVADTLKYVVQHQFRINRFITRINDFYSNVMFLEVFSLHLVFSVATFQISQEGLTTMRYGMIPLLLIFLAIFTVCSWTANDVEVKSAAVEEISSHASWWTWNSSNRIVGQCVSEFANFKLHLNAGLFNSMSLDTFTDVLKISFSYFLFLHSLHVAQRKAD

>EdanOr12P

LYLSRFFILGINLWHEDIFAGHLRHQFLRFQKLLLTLYYATYVIMLIANIIHYLFIDWHKPFAKNVNILVLIIRQSYGIYKLYIIHTNRHTVHKLLKETFYDVAAPICLRPLINNYKQLFLKKSKVMSFTSIYLFMIFIPTVLSTILPFLNYKDIELKMFNFEIPQIIPKIMYALKPLAVYPVLVVTLGINVFYIALLTVAQRMLEHLREVLNFLHHGIGTREINVERIQNSQQEYQQRVAAMLKYVIQHQSKIFRFIEDVNDLFSNVMLLEIWTLHVLYCTFILEIVATKRLSTAQGAAMIHLAATYVLIFAICCWAGNEVKVKCNIIVQESATHVAWWTWNTENRIMGQCIIEYAGYKLRLIAGSFYSISLNTLTTVLSASFSYFLYLYSLYTSE

>EdanOr13P

MNRQNRKLDKNSHQFNLRFRFVLIINFLFGHWFYIAPLKLRGIYYVSALFFVFTINFFSISMTIIDISNLYRATPNFTLVLYFSLGVLASCRSTFQTIYVLIKRKSFEETIQRLNRILNFKELEDZQFKAIDKAMKFTYRIAIFFCIGFTACIIMFHLSWWIQTESGGAISIELLVEVCTITNVIKIGDMLRHAILYFVAFLVYAGKVIAMDIMFLSWYSLLVEQMDLLVEGIEKSLHQADVKTARRSLTSWLHFHHELRRVMQKVNRVAAPCIVLIVVVNTLQMCLLVFFVGFVNSLSAAVLLMFALFAFLQSFYYCHFGQKIRDRLIRLQDAVYTSPWLGSRREIQHAALMICNAATERAMPLPGAPFFALSLEFFASIIGAVFTYFILLFQLK

>EdanOr14

MSPFSVYSFLKYIMRGCNLSSEIQHHFRFILVLNFVFGHWFHVSPLKLRGVAYICAAIFITLVNLAVIVRATFNVHNLIHALPHFAPSLLISLGVIGAVQSVFRTTVVLLKRKEIENIFLKLKVVVDGHIFPDIQTKTIKTALKYSYISVSIYIIGFITAMIFHSFSWQMQDDFIGRNENTTTIKSIIKHNALISKSGYWEQLTYTILYYTVFLISVGKIAATDLLFFSWYSLLIAEMDLLVKGINQLLCQPKSNTMHCNLSTWLQFHQEIVRLMQRVSTVASPCAVLTIAVNTLHMCFLAYIVTKRYVDTLSMTVFMAFALLALLQSFFYCNIGQKIRNKVAELQQAAYAAPWLDSPRDTQHAMVMICSSATERAMPLPGAPFFALSLEFFASVLGAVFTYFIVLLQIN

>EdanOr15

MGGYRFTRNDVALKFRLVLSLNFIFGHIYPCKLGGIKRVIARIYIIFINFFLFVMTLLTINNLINAFSSFLVALYIFVALIGISHAAFRSAYIIIKRKNIRMIIYQFRRLVNEETFKKSQNKAIQKAFKRSYRTLFIDTIIYCLCLILHLILWIVKNIDTDEDETGLSLREVVIKYNPVSYLSGINTKRLYCILYFITFFISIGKVVTTDLLFHSWYALIIEEMDLLMEGLKVLIRQSEPRVAQRNLNQWLLFHHQFMRVMQKVTTLTSPCIVVTIIVDILFVCILVYIVVKGIVGAFGMTVLLGFAFVSLIQAFLYCRIGQEIRNRIIKLQTVAYSAPWLGSPHNTQHAALMICGAATERAMPLPGAPFFALSLEFFASTLGAIFTYFLVLLQLN

>EdanOr16P

MAYVTVATTSSKTYKSTNRIHLVLNFIFGHWFHVSPLRFRDTSYILAYIFVTAINVTTIGFSIMSIYNMILSARDIKNAVFLAAGVSSMIQSAVRSLLLITKNNNXKLTNKVSGYTRFIAIFLTIVVSIMNILGFVLIGVMYNWNKSTDAVNNEDTLDFQEVFTIFDSYVHGSSATVFILVMLCSWLVTSFILGKIVATDVLFCSWYMQIVEHYKLLVASISTDLLPSNNETAESITQWMQMQHEINRLLTKVNSITSPCIMLTMVGGTLQICFYAYLVAKNAVIAMHAGLLIVYSFLTLFQMFVYCNFGQKIRNQVARLQDAAYSAPWQRSRREVQHAALMICSAATERAMPLPGAPFFALSLEFFASILGAVFTYFIVLLQLS

>EdanOr17P

MQKLFYRAVYKNEPSIIPTWSKPVQLGYGFTIVTILGAVFGEIFPISPLQRRHLHKLLYFMIKTYTILINLILIVFAISIYYGIVKYLQSFSSIVYSASGIFSVTEGILRIYFMFINRKRIQKFVLQLKSFINIPEHRSFQSAITSRISIGIVILILFVFVAYKLALVQLLXSVNFEALKSLKDNLTMSNAASNTSNVPVELVEFYLYMDGMHRLIPEMSINVSAGLVYAFCLMGVGKVFITDVFFLACYWLIGEQLEILTCRIIPVIRDANREQLASWLDYHQQLTQILQDVNGATSYPFAVGIIFSSLQICVLTFVIIRQNLDLTNSLSLILFNTVIIWHLFSYCIFGHRIRSKISRLQAAVYAAPWLGSRRDAQHATLMICSAATERAMPLPGAPFFTLSLEFLASVLGAVVTYFIVLLQINE

>EdanOr18P

IFKETKKRSKDDVLFLFSKENDLGTGVRTISIVCTIFGFWLPIKPTFCIRRCSVSYILNRLYTTILCVCLMCVTVTSAYAVITSTHRFATTVYLFSAFFGQMEMSVRPLFLLFKESQIKKLIVTLHSFINEPLLKNLQAAVIKKLSRYIHCMLTFEVFLFIISMIASFLSTKVEIESVSLDGIVTNFTQVGLSPEIAEIRAYAEVFFSFMSQSIFEYCMYMIYMVCFCGLMIRVSTDCFFYACYWLVAKQLELLADNLENSMRPPTQLATWLRLHRDLNRTLNSLNSTSAAVIITSVVCNTMVMCCVTFAIIKQQMATPNILSMLVFSTTSLLQLFIYCLMGQKIRNKVTRLQEAAYSASWLGSRRDTQNAILMICNAATERAMPLPGAPFFTLSLEFFASIVGAIFTYFFILLQMNDTQS

>EdanOr19

MAAIRRANEIGLGIGFRVLGILSIVFGQLFNIHPINIRKRSIAYYVTHAYTFIMVFYSSFITIGGLILILKNSSNFSVVVYYSTGFLGVAKCCLRSYFLILKKRRIELFTENFRRLLNESWLKSHQRRIIFRLTRYILILYAFLMSFYVCAMITNFNALITRIKHANETNSIAVPQFFKDATNEVMNNMWLNNSNISRFFDNLIPISISMVLVYSVAFLCIGKLITSDVLFYASYWLIIKELELLISKLKGVIRRSSTEELTLWLNYHHKLEKLLLEINNITSLPVFVSVIFTAIQLCFVAVVIVKRFLQDSSDSISLLLFAITSMQQLFMFCLLGQKIREQLVELQRTAYSAPWLRSQRGMHSALMICNAATERAMPLPGAPFFALSLEFFASIMGVVVTYFIVLLQFNKE

>EdanOr20

MNTNRLSVVLSFMFGHWFYVSPLRFRGVFYIFAWFYTITVNLSCTLMTFVCIFNFFMANRDFKRIIFLATGMFGMIQTITRAIFSLFYKKQIENILELFQNLLNNSEYKYRKVKLLFKVSRYSKGIAAFLILIYLTCFLYGFIIFGVVYENGRGNTTIDIFDENITDVREEWAKVDGIIGNIPSNLFTLYLSFIYLTTNWGYGKIIASDVLFFSWYMQIVEHYKILCYNLSSILLNCNKLDTEKMKRWKRLQHELNRLLTRVNTVTSPCIVVSMVINTLQVCFVIYLVAKGVLDVILAGSYIIMAGMALFQMFTYCHHSQKIKNQISKLQGIAYSAPWHNTQHNTQHAALMICSAATERAMPLPGAPFFALSLEFFASILGAIFTYFIVLLQIN

>EdanOr21FI

MVQVASADGKKMLSYNKKNVLDTHRLPMVSNFIFGHWFHVPPLQFKGAAYMLAWSYIILIHAIFVMFCAAGIYNLISVATSGFKVSVFIITEMSNLIHSNCRAVFVLFRKHEIEEISTRLIKMRQRSNVTSNVARNISIIFTFLTLTYSLTVGLGLISFLTQYKFENITSANENEIYLKNAYSNMNSNLKDSPIYIMILIVLVAFILSNLGLGKIISTDVLFYSWYMQIEDQYKQLTMSLGMAFLHCNHGHSDSAAHWIQTHREINRTHKKIIELIIEINNLQVAEESVAYLVAKSSKLQEASYSAPWLGSRHDTQHAALMICGAATERAMPLPGAPFFALSLEFFASILGAVFTYFIVLLQMK

>EdanOr22

MVQVASADGKKLLSYNEKNILDTHRLPVVSNFVFGHWFHVPPLQFKGAAYILAFSYAIFTNAIFVMFCGAGIYNLISVATSGFKVTVFIITELSNLIHSNSRAVFVLIRKHEVEEISTQLIKMRQRSNVISNVARTISIIFTFLILMYSLIIGLGLTSFFTQYNFENVTLANENEIYLKNAYSNVNSTLKDTPNYIIILIVILTYILSFIGLGKIISTDVLFYSWYMQIEDQYKQLTMSLGVALLHCNRGNNDSTAHWIQTHREINWLLARVNSVTSPCITVSIVVNTTQICIVAYFVAKDTLALVHLAALLIFASLGLFQMFMYCNLGQKIRDQTVKLQEAVYSMPCLGSRRDTQYASLLICNAATERAMPLPGAPFFALSLEFFASILGVVFTYFIVLLQFN

>EdanOr23

MITNRLPVVSNFVFGHWFHVSPLQFKGAAYILAWFHTIFVNVISIMFCMGCVSNMISAYSFGFKVTVFIATGAFGMIHTLSRAIFVLTRKRKIEEILVRLLIIRQRSKIISNITRNISCLFIFYTVTYSFTIVLGFVAYEIEYKTRNVNDTNENIIFLQEAVSNVNAMVEGMPNYMADVLIFITMTITSVFGIGKIISTDILFYSWYMQIQDQYRQLTKSFAKVLINCDKGDMESATHWIQTHREINRLLARVNSITSPCITVSIVLSTIQICLVAYLIAKNTLITLHLAALLIFASLGLFQMFMYCNLGQKIRDQTAKLQEAVYSMPCLGSRRDTQYASLLICNAATERTIPLPGAPFFALSLEFFASILGAVFTYFIVLLQFN

>EdanOr24P

MKVHVFGALTEKRYVNSNRVPVVLNFIFGHWFNVPPLRFKGVAYILACIYTLIVNANTLLTAVGVIFNFFGGKKESFQVFIVLAMGMFAALQPICRAIIFWVKKKEIEMVLMEFQTILIVLQQPQLSFKSSRKTLHLAIFLSVSYIIAGIMSFVLFAIYYENDNSKESKDEESTKYLKDVWSSINIISRDIPTHILAICVTLVCISTIPTVGKIVATDVLLYSWYIQILDFYQKLTENLATVLMHHNDHDAVGIAHWMQMQREINRLLTRVNAIASPIFVVAIVLSTTQICFVAYLVAKNSLIILHWGSYAIFAGLGLFQMFIYCSLGQRIRDHSKFQEAAYYSATZLRSRHDTQHAALMICNAATERAMPLPGAPFFALSLEFFASILGAVFTYFIVLLQMK

>EdanOr25

MLQSEGVVPFGVRVMIWCNRISGFWLPIPASPWRRLLLLAFESLVLIANALSFLAHIVRIHTDKIDFIVYMLGIASLMKYAYIVCASTFLLLYKKQVEQIFKDAQNLLKNRQFYYYQMSVLNRTSKRLIVYLSLPILLFFVIQMFLATNSARILRLSKMPNVTSEEDKFVESILRNDGTTLDYSLIILTFTTNVLTQFKAIALDAMFLGLLSFVSEQLGVLKITLHEAILVGPSPSYKRTDLDAWLNFQYRLARLTGKINATWSFLVVVLFFCTALTLCSLSYSAVKLSPLGIVTYVIIAHGLISVLPLFLYCHAGQNLRTQGEALAEMAFRGPWLHMKPALMPGVLLLALDCSRSFVARGGPFFTLSLEFFASVVGAVLTYLVVLLQMK

>EdanOr26

MLTIDNHVFLGFRMLTLCNLITGFWLHTPSSWPRRFLVLIYEFFTICIVITSIFIHSLQISSNEDFFVLIISMTATLKLTTIFVFAVTLLVQKRSVLKIICETQNLINTYKFRFHQNTVLQRTSKIITIYFLVPFLFLIVNNFHAMFSMKMIEMLGTNESNVTSNEKKFFDALIHRDENDQNRDVNNMILFLFAFSQTFSLAKSITMSATFLGLLYFVGEELRVLRLTLHEAMLTGHVTSYKRIQLVDWLRYQYRLARLMGQINSVWSYAVAVMFACDTVSICFLCYAVVRVAQYISVIRVLISYWLLAILPIFLYCQAGHRLRTQADALVEATCRCPWLRVTPSLVPGLHLVALDCSRSFVARGGPFFTLSLEFFASVVGAVLTYLVVLLQIK

>EdanOr27

MLQGEGIVPLGVRAVVFCNRLAGFWLPIPVSPWHRLFLFVFECLVILINAATLVVQIIKLYHVQNFVIFLIWIGALLKYSYTVILSIYLLISKKNIEQIFKDAQGILKNKHFYYYQVSLLNRTSKRIVIYLTIPFLCFIALQICSITFTNSIMKISTTTNVTTDQDKFIKAMFLNNGTFFDQALIILTLISNILSQFKSIAVDAMFLGLLYFVSEQLNVLKITLRDAMTVTGKVSSFKRTDLQTWLNFQSRIARLTNKINATWSFVVVLVFICTTLITCFLSYVMVKLPQLGIVTYISLAYWLLSLLPIFLYCHAGHRLRIKGEEITEAACRGPWMQMNSALVPGVHLIALDCSRSFVARGGPFFTLSLEFFASVMGAVLTYLVVLLQVV

>EdanOr28

MDDVVPKGLRVMFFCNKISGLWLQLPTASSKRLAVFCFEISIAAMLVCDVIFNIVHLFSAKNFVLFILSTSGILGMTHTTFSSIMLIINRRNINNIIKEAQHLIKLPTFRYHRSIILRRVSTKSIAYLMLPILGFVVAQINTIYFTSYNVLNPTTNVTSAEEQFIKDAMESSADSEMNSIIKILLFMNFLIQSLASFKLIAMDMLLLTLIYFVSEVLKVLRATLHDAMIVGPVLSFKKVDLNTWLYCQRRLSGFISRINVLWAPMIVVTILCNTLTICFLSYSIVKVYEIKIFMWIVFGFTMMTMLEVFIYCEAGHQLRTQGDAITATACQGPWLNMKPSLVPGIHLVALDCSRCFVLRGGPFFTLSLEFFASLVGAVFTYLVVLLQMK

>EdanOr29P

MLTMNGVTPLGLRVVVLLNKMTGLWLQVPSSRSQRLAVSVFEWIIVVVLIFNIIFTFIRLLSIKNFVLLILSISSILLMIQSTFSSIILLINKRHITNIFKEAQYLIELSNFRHHQAILLRRVSKRSAFYLIFIFLAFVVTQINSLHFTATITINPTVNATNPEKQFIEEAIGNNGNEMNILMMISFFSQTLAIFKLISMDILLLILLYFMSEELKVLRATLHEAMLIGQAPAFGKVDLGTWLSLQCRLSRLLARINKTWAPMIVVTILSHTLSICFLSYTIVRXFKTLVWIVAGYTLLTFLDVFFYCEAGHRLRTQGEAITEAACQGPWLQMKPSLVPGMHLVTNNCSRCFVVRGGPFFTLSLEFFASLVGAVFTYIVVLLQFK

>EdanOr30

MLSMNGVVPRGLRAMILCNQISGFWLQSPTSRSKRLLVSIFEWFIVILLIYNTIYNIVRLFSIKNFVLLVLTTSAILGITHTTFSSIMLIIKKKQIISIIKEAQYIVKIPNFYYQQLALLHRVSMRTVYYLIFPFLGFVFAHSNSLYFASYIVINPTINATSPEEQFIKDAVANSDDDINIMQILVLINLFSQSFSALKLVCMDTLLFTLLYFVSEELKILRATLYEAMLIGPVPSFKRVDLTTWLHCQRRLSRLMARINKTWAPMIVVTVLCNTLAICFLSYTIVRVSEFKILVWIVIGYTLLTFLDVFLYCEAGHRLKTQGEAITDAVCQGPWLHMKSSFVPGMNLVANNCSRSFVARGGPFFTLSLEFFASLVGAVLTYLIVLLQIK

>EdanOr31P

MESVKIMKTPIGFRAVVVSNFVTGFWIPESPLKFKSTQVITRYAMKFYMALITVSLTLHVFVQAKVVLKLQLSQKIAMQLILVISQITSSIIIAYRILSIELNRRSILRVIKNIZQLINLPYFESFKNSLTNRLSNRCIVYLSLPAFGFVCFQIYVMFNFHLVQILASTDPNVSNFTNTSTSKVVKTAFSDISLNDTLQGILIFITTWIQVVVIAKNIAMDALFLGLVHFVAEELDVLRRTLHEAVLVENSAIKFSSCVHLTAWLEHQRKLARVMELINTVWSPHVVAIFLNSTFCMSLVAFAVIKALEHGISMALLVAGFLLLNMLPIFLYCQAGHHLRTKGESVAEAICRGPWLCGRCRLDLVPGLHLVALDCSRSFVARGGPFFTLSLEFFASVVGAVLTYFVVLLQIK

>EdanOr32

MVAFQLCLEDLASKMWENHEDDLKPFGIKTVHIFNLVTGLWLPLTQFNNRIVSILIHVYEGFLVVFLASYIVVEIYTLTTTIDFEDAFDLIAIYSVVGIIVFSSLCRSVYLLLFRKKLLALFQNIRKLCNQRQVIHHKHELINRVSKNFLKNTIFPAILLLILHLSSLIYYSSHLREEPFRLDGNATGETKSVFQKLRELGNKLEGTWMYYGWVFGMSSQFIANMKLIASDALLLGTVEVLGEQLSILESTLDLTLHHKDFLFKNFSFSIQGWIALQRKLIQLTEEANAFWSPIVVLTFTCNTVLLCLNAFTFVKTRSSSVSTIPIILLGYLVVNLMPTVIVCHAGHRFRRKGHAVSDAVRRCDWLRAPRALVPGLSLVAADCRDSFLLRGGPFFTVGLEFLVSVVGAVLTYLVVLLQFK

>EdanOr33

MLHAAYSKRPLGLLAIQAVNLVTGFWIPLPPFNFSSIYTPYFVAYEWILNLGLIFHLLYSQLNDIVSYTEMLSKDLISFLGIYVYTVAFIQSTIRSMLFSSNKSKLIKILESLQSLIDRRIFRQFQAKILKSSPNKPIKTIVIIFVGYCIIQTANVVNLFMNPNYTTKITHHNSSDFDARVYMLQQKTREFDFFLKTVFNTVVATISILKLLSTDVLMLTILNLVTEELILLKNSIKNAIYVNNSNKNIIRNNDNLDLGAWIDFQQTLARILGNINSFSFVYTSIMIGINASNMCLMAYICTKLVSHSSLIVAVLAGYCFLIVIQTFLYCEAGHRLKQQGEGISEAVCRIPWLRTQPYLVPGLQLLVQDCSRSFVVRIGPFFTLSMEFFSSLIGVVLTYFIVLVQFN

>EdanOr34P

MVKSVKICGSTVTKTLGFRAVLYTNLISGFWIPLPPLNFKLKFLVKCIQIFEWFIVLSLGTQLVLTTYFLSQNHIFTWNSILRIVASIQNLFHSVARSLIFANSKKQLIQIIIDAKKLTNRRMFKSLQGGVILRTSKAILRALILPLTLLAGGQFFSXLYKLIEAWLEATHLERSKWPDFVLSTFASLGYTISYIKIMSLDALFIGLFHFVAEELRVLQSTLFDAVHATSKNDNCNKKLDFVVWTNQQHALTKLVENINAMTSPIVLLTMLGNTLCLCFLTFISARHAIRNESILEVDTLTAIYCVITLVQTALYCEAGDRMKKEGLGVSDVACHGPWLRANPLIVTSLPLLARDCRRCFVVRGGQFFVLSFEFFASVIGVVITYFLVLLQMK

>EdanOr35FI

MVLKSRILKSVRICTSMGMDVFYPARALDIFGLYGKKKYSFGKVMGRGLMVSMFILQVFFQWKVMRHMEHTVITILYSFYLSFVGINTVFRFNFFVLKSVYIYVMQCIVLAVTDAFICSLMLAAAERGAFLARVAPRALLPGRDGEVTRMCRAWIRYHQHYLEILKKINKEIGPLLLQMQIFSIIRLLFNTFLGVVEGKVLILPSLSDSAPLLINLLMFSVAGQKIINASNRLARMALSQEEVSRGALESGPTRNVLQIVRARCAVRDGKITGLGLFVVSIGFFTRVLSATFSYILVLYQLHYKKT

>EdanOr36

MGLDVFYPAWALDFFGLYGKPRYNWNTILTYTVLVSVLMLHTLLLLRVQFLEYTVVMTVFSICLSLIGTNFVFRFCFFVYKHTPHNECKQFDALYSDMRAQSSRTIRRFCRTYFTYTAFCSLLLVATQFISSFFYSERYRFDADNKYFYVDQWRPLLYEIWLPWGDPHEWPYYPYVLLWQEFVYLMQSCVNAVTDAFICSLMLAAAERGAFLARVAPRALLPGRDGEVTRMCRAWIRYHQHYLGILTMINKELGPLLLEIQLFGITKVMFNVFLGIVSGEIIAILAIADAGPLLINLLTFSIAGQKIINASNQLARVTVNAAEIARGGLQLVSLRNLLQVVRARCAATADGCITGLGYFKVSIAFFARVLSAGFTYVLVLNQLKSKK

>EdanOr37F

MDVSYASNTLKSFGMYGKTKMTFSVLLTYVTLFLFFSFNTLMVVRLLLNNYTILMSTFIFYLVIIAFNVLFRFYFFALNNVTPRSVSVNFDVLETEMRIKSNQKLRKFCRMCYVFGAVCNLVFVSSPQLLSLTYDQRCRVDVEGMRLYLDQWHPLLYEIWLPGTDAHEKPLYPYIYVLQSFTIQLIIDVVTYAFLCSLMLAVSERGAFLARLAPRALRPGRKGHVTLMGRAWIRYHQHYLGIIRRINKELGPFVLLIQVYGVTKLMFNVFFGLVAGEMMIALAASDVCPLILNLLAFSVVGQKIINSSEQLARSTVSVMEITRGGLKPGPTLSALQVVRARCAMGDGKITGLRLFTVHIGFFAKVFSAAMSYILVLIQVQHHK

>EdanOr38F

MDVAYPVRVLRFFGMYGAARKNTFFTFIFVLLLSIITVLMIYRFFYFEYSMMMYIFISYISVITLNIIYRFWFLVAPPYIASQNFSELEKKMMAKSTRNIRRFCLFFYIFCALCNIVFVFVPSISSIFYKERFRKDTEGIRLYLDLWRPLIYEIWLPYFGNPHEYPVYPFVLVLEVSAFWMQAVIDVVTYAYLCSLMLAVAERGAFLARLAPRALRPGRKGHVTPMGRAWIRYHQQYLEIIRRINHELGTFVLEIETYGVIKLMFSAFFGKTSGEMIATMVASDTGPLLLNIFAFSIVGQKIIDSGNKLAKSTISVAEISRGILESGSTRNTLQVVRARCAVPDAKITGLRLFTVSIRFFVKVISAACSYVLVLLQLQSKKI

>EdanOr39

MLWNTAPVRVLHAFGFFWDRKVSLVLTFIFLISHMTTHVLLLINMRQIKVNLTDACTIALIELRAWHGIARAFHILLNRRGIFDLVDFMNNSIDPNNKNFLAMETTIRLRTSRIIRKFCRVVFSYKFFSHIFFLLGFFSGIMKPERYVKGTIYNDQWRPLVYEVWWPWGDIHEFPNYYYMLPLQLLMHFVHNGINTIMDLFLGSLMFLVAEQATFLTNTASRALRSGRRGQVTSSCRAWIRYHQYHLETIRHLNSELGQQILMTYASTACQLMLATFLFRELRHYPGTMIIHSIFVITPVLLHLLVFSMIGQRVINKEASLTSTVLNTVEVSRGELGPCPVRYTLEVLRARCAVARDYINGLGVFTVSMEFFTQTLSAAVSYFVVLIHLKT

>EdanOr40I

MELQFILEALKILGIYKDKDSSFIFRLCRYVTIIQFLILHVFVGLQFISATVYIRVNIIYYFLVAATVIGGPLYISRCGKRIRYIIDLLEGDLPGITNYGEMMREEIRMKFRSERYIRRYCIFVFSHTIVTMLIIIGSFVIFRTIATMIGFTSIITNSSQGQSTIPRFLMYVMWWPTNIRELPTYYYIIAWQSFNGVYTIAITNVMNAFTSSLLLAASSRAEMLAKTAKTCLVPLRNNPKRFSKKFRTWLCKHQRYLTFNDTTMSATIIIVMLEKILQMAIMSYAGQKVIDSSLLLSHSVVSSSQIDTGVLSASRNTSALQVLHTRTSGPISKVSGLGFFTVSMEFFASVLSASTTYLLFLLQFKAVGGPTAYEVPKE

>EdanOr41

MRESSNMLLFNKLCYEMNYASPNITCSLALDVMEVDLNLTVLRFAGFPDLRKSNSTRRTWFWINLIQFLIPHILMVIYYLPIGLNVYSGVNYFFYASNCIFTITRWFYFMRNNNDINIILNNICEKEVNGSVTMMKRKVRSRLAQRIRRYNAVSFSYLACCAIALIGGFILYKVKSPSGTSLKPPRRILLFGVWWFTDWHENELTYVMLFLWEGVVLMTSLAASIAVNALICALLLAVSARANILSYAARTTLMPNKIQRTKRTLSAFRSWICKHQRYLSTLKRINIVFGPMILLSHFEAIISIIAFSYMSIKVRDQVMSALCLAPVFGSLLHIFLLSKAGQEVIDMSVRLSGSVMSIAHLQSGATVSKRIRSILQVLTARLSVINDRASGLSYFTVSLGVFTSILNACVSYLLVLLSFRGPENEPEYFAKKPI

>EdanOr42I

MEFEFNLKALKVLGFLVPTDKRPSCLHRLWNVVTLLQLVILHALIVIDFVAGLSPTMYARVLSSFHMMLGTIVVFRSXXXXXXTIATFLRVGSYTMNKKSKGTIRFILYITWWPTNVRETPTYYFIILWQGFTATFTLAVLTITDAFTCALLLAASARAEFLSRTISRALQPNRYNKHQLSRSFRAWLMRHQRYLAMMEQLNVVAGPMVFIAHLTTLLMCIVFSYVIYKISDTIMAGMLMGLNLGALIQLAIMSYAGQHVIDTSARLNCSIMHASRVQQGLLSRGPNRSAFQVLMTRTSVLNDRCSRVSGIGFFTVSMEFFSSVLSASMSYLLFLLQFKSAGPKVIQDLNDK

>EdanOr43

MDSWLQFQINLRALKIVGIFIPIHTWEKPSKLLQTWKFLGIFLYIVLHLLVIANLLLESKTLGIFSRLMSSYNIFVCTIILLKIVYFSMNSMRIRKLITLIDEVTAGRTNGWRLRGLSLREDLYMRRKSARNCRFISMYIFLVTIGAIFVFIGTVMLFRTITIVLDWSKPVNENKQIIRKFRRVTLYHMWWPNKIMELPTYYFILLWQVFCSMISLTVSCTTDALTCALMLAASERADMLAKTAPRALVPTANRNTLAPAFRAWIRTHQRYLKMMECLMQVIGPMVFISHTMAFVTFIIISLGVFKASSNSIGDSIMVATMLTITVRGLIQLTFMSYSGQKIIDSSARLSRSVVNAAQVQRGLLSRGPNRSALQVLVTRMSVLGDRYAQVSGLGFFTVSMEFFSSVISGSLSYLLVLLQFKSIVSTI

>EdanOr44C

METDFILRTMRAVGMCVTNSVKPKFSKTGIVMLFMFVSIMEVLMIAGYLTAKSQSLYSKLMSIYHTFNFVVTIFRVVYFSTRRFHLHFIIELMEDFPVISNPCILFHRTFLIYLQSIDASIVDAANKPVKRYLISYMWWPTNIRKTPTYYFILTWQCFAALYATFIAAVSDAFTCSLMLAVSARAEMLTRTALRAIALSKPNVRLTAKFRYWLQRHQHYLKLVKMLQQINNLTSPLVFVSYLHSLVTCIIAAFIIMKDNNETLAMVAGTYALLGTIQLALLSHAGQNNARLSSCLLGTITNEVLSRGANRSSLQVLMARTSIIDDRFARISGFGFFTASLEFFSS

>EdanOr45

MDTSELMFPVETLGWVGFLAPHVKPLSRRSFTWSALTLLQYLGMNLLIFARLASVDFSLSLVISLVAHTVNLVETIFLRIYYTIRRDAISNLVQDISAVSEMNEPFASADREMRNNSRRRIKNFSSSVFLFNFAVALLTVLRYMFSSAVRGLNECMPENDCRNTEGANDHHGRLLFDIWWPGNPSVTPVYYFIMAWQLATLFTHISNVAASDAFACSLALAISERSALLTKDAGCALLPGHDNQVTPFCRGWLKYHQNYLRVVVTVNDVLGPVFFFTFCIAGLNVITLSYISAKAQDSGLLTSTVTVIAVLLVRLFALAYAGQCVHTKSEELSRNSLSAAQVAKGALKDGPARTTLLVLITRLSGGIKPEEISGLGYFAISLEFYTKILSITVSLLLILLQLQQ

>EdanOr46

MVIETIRTQLHSSEWQREHETAEVLVGAHLVISPAFMTPSNVTLTAAFVSNSLQTAMRHIYFFVRRKQIAELVENIRSSPSKESVDVGAAETAMRLTSATQVHHYSAVIALHHACAAAVSSVFIFILPTVLQSSENPPRRPLYSEMWFPYDLKDPLVHTYVLFWQALAVLLNVASAATTDAFTCSVMLVTAERSAMLARVAPKALLPSQGNLTPHFRAWVKHHQHYLGLFSRINTLMGPIIFSVHLFGIINIIVFAYLIMKVDLFSMLVALAHLASVLLQVYTFANAGQRVIDNSEELGRNTLSAAQVARGGLCEGHARSALLVLVARCSGARPEELSGMGYFTVSVPLFSAVLGASVSFLLVLVQFK

**43 *Thermobia domestica* ORs**

>TdomOrcoFJ

MKYIRQGLVADIYPIIRTMRFVGHYILRYYQDDGPMKRAFRAIYSVSNISLITLHFLLGAVSIMFKMNDIEGLVANAISTFFAFHAVTKMIYFAVRKKAFYETLDCWDVTNSHPMFAESNARFKMSAIRRTKILLLSVSGGCALFIIFWSIRPFFVAPFRTIMDGNETIVVENSPLIVDAWYPWSLKDFTFFAASYFYQLYWLIFCIFQVNSIDVLFCSFLIYACEQLKHLKEIMTPLVELSAGRDPEALRKAELWPEITAIDKSASRLDGTPPPYQTATRNRIYPETLGVDMERSLVLSDFAHLKEPMVTYSTDEANIGENVLTKKQQLYVRSAIKYWVERHKHVVRFVESVGDTYGLALLLHMLTSTITLSLLAYEATKISAFDIYAMNVIGYLLYTLLQVFLFCIFGNDLIEESLSVMKAAYECPWYNGSEEAKTFIQIVCQQCQRALSISGAKFFTVSLDLFASVLGAVVTYFMVLIQLK

>TdomOr1J

MPEPQKGLIALLQTHIKFLQFSGHFMLDFHSNDAPMMRWFRAMYSIMQILISTLHMIFCVLRILYSLSNLSKLVPVVVSTTFAIHGVIKLIYVAVRRKTFTKVLRLWDDAGTHPMFEKADEMTLQVTRYRTKRWLIISTVFYLFYTVFWTVSPFFDKDYEDIIVDNETMRVDKPRLIVGAWYPLDLTSSPGYQIAFLYQTYWAFFGPMQVHSIDILFCCMLVHASEQLKHLKKILIPLVELSSNPDGKSVTEKSKYMGVSQLSLLSEGNNYMSELPRRRQMAWSTNRIYVDEVLNRENVMNSGGGNTPMDAGGAAELIENEKARHLRSAIKYWVERHRQIMRFASDVEEMYGLALLFHILLASLTLCLLAYEASQIREMNVYSINVLGYMIQNLLHIFVFCIEGNSLIEQSSSLMRSVYDSSWYAGSEDAKVFIQIVSQQCQRPLSISGAKFFTLSFDFFGSVLGAVITYFIVLVQMK

>TdomOr2

MKLEEYKAHGVAELLSPLIYLMKYTGYLFLFEKDNKRVKRKWKTYFQVAFSLLLYVLITLHIASFITDAVLKSNDFRQFIRSFLPTMSALTGYYKLYYVAIRRQVFYKALRSWKEGLKTHPLIEDLEDRTIEYTYRMCKKLTIGGLILVYIVYVLWSLKPFFIVEKTSVQRGNTTVEVIQRHLIIDGWYPFDISSMPNFIAAYIVQLIYAWFVCGQLIAFDLLLCEMIYHLSQQIRMLKRLIAPFALLDSDQLSEEVDDLVQMIVQNKLNHNSQRLKLDSLARNLEKGEAKIQLSEYKLFIQLSKYRSELLPEMRKDLMSSLIKYWVDRHRSIIRFTDSVQETFGTALMLQMQNSVLILSLLAYEASMEKQFSMYSLAIIGYIFHCLSSVLLFCYFGDQLIQESNSMLRSLYDSAWYHTSRDFKHFITIASQQCQWPLKITGHMFFTLSYEFLASLLSVVMTYYLFLIQLN

>TdomOr3

MKLEEYKTHGLAELLWPLIYMMKYTGYLFLFDKEGVKVQRKCKTYFHIVFSLLLYILITLQTASFIAYVVLKSDDLKQFIRGILPLMNIITAYYKFYYVAIRRQVFYKTLRSWKAGLKTHPLIEDLEDRTIQYTYRICKKMAFGGVTLVYIVCIIWFLKPFFMVKKIIDQRGNTTIEIIRRPLIVDGWYPLDISYTPNYIALYISQAIYAWLICGQMIAFDLLLCQMMYHLSQQIRMLKRLVTPLALLDSDKLSEEVDEVVQMTVCNNINLNRKRFKVESLERNLKEEADIQLSVYNLLLQLSKYRSELLPQQRKDLMASLIKYWVDRHRSIIRFTDSVQDTFGISLMYQMTIAVLILTLLAYEASTEKEFSMYSLAIIGYIFHCLATVLLFCYFGDLLLQESNSMLRSLYDSAWYHTSRDFKHFISIACQQCQSPLKITGHMFFTLSYGFLASLLSLVMSYYLFLIQLN

>TdomOr4

MHTKFKSRGLVAIVWPSIRVLQISGHFILDFYNDNTFSKVLVRALHSIIQIAFNALHFIFCFVDIVLKADDFEKMVGTVATTFYAVHGMTKLFYVVFRRKLLYHTLSSWNGLKEHPLFWKSGLEACKRSYVTSKKLLYYVNGFILIDAVFWAAKTFFSYNLEEVQVGNSTVTIKTKRLIVESWYPWDIKGSGFIAAYIFQYYWINVCVIQVTTFDMLFCSMLVFAVNQIRHLKTTLKEIVELGCENNEERETKIIDQWLDTYNEVDNSNFTSIEQHNMEMIKSFRNGRNKLKKKVLIQSLIAYWVCRHRDVIRFVTAVDNTYGLPVMFHMLLATITLSLVAFEASNKQDSGIYVGNVLGYLIHNISQVYLFCAYGNKLLEESSSLLRTLYDTPWYETSKEVQTFLKIVGQQCQRPLIFSGAKFFSLSYDFFSAMMGSVLTYYLFLVQIKNER

>TdomOr5J

MGNQSDYRRYPGLPGLYWRSVRLFQLGGHFFYDFHTNYNFWVVILRIAYSVFSISVVTLHFILCIVDIFYNIVDFRKFLSTIATTTYAFQGILKGWYSAFHIKSFRDLLHSWDHVQTHPLFAPDNEICERTASRRAKRVVTIVFALIVFDANFWIIKPLFEYKLTEVDTGNETIIVKEKRLVVESWYPFNKAENFVAAYVFQTVWLYCCLLQVTSLDLVLISMTIHAIHQVIHLKHTAKKIIYLSSSLKVPEDYNHLSSDESRYNSRKDDQGRQYMFSAVADIQLDLVEGLGDLRSTRTKLVYSLIKSWVDRHREIIRFVDRMEETYGLGITLHILVSSVTLTAIAYEASREGNSGLHVGALLWHFIHSITQVFIFSYYGDELMQTHSNLIQDLYNAPWEDATRETQMFLQVVSEQCEKPLRLTGTQFFTLSFDFFGAFFGAVVSYYLFLVQFK

>TdomOr6J

MKKNTDYKTRPGLAGLYWRGIYLIQVAGHFFYDYHQNYGFWRMLYRIIYSIFSITVSSLHLIFCTVDIINKRADFRRMINAIATTTYAFQGVSKMLYLAFRPKSFQNLLHCWDHVQTHPLFAVDNESCDRTAYVRAKKIVTLVFTLIVLDATFWAIKPLLEYKEKELTEGNETIIVKEKRLIVESWYPFDPAESRINFLAAYMFQVVWIYLCVTQVTSLDMLLCCMVIHAIEQIEHLRNAARIVVNFSTKYPQQKNNFFLEELAYRYRKEFSQLQQPSNQGTQQLVSTSPASAELGLIGNSDSDERNKLVFSLIKSWVVRHTDIIRFVDIVEYTYGISLALHMLVATLTLSVLAYEASMEETLGIQVGNILGYLLHSIGQVFLFCYYGDILMQSSVNIMQDLYETSWDDANADTQMFIKIVYVQCQRPLKLTGYKFFTLSFDFLGTFMGAVMSYYIFLVQFK

>TdomOr7JC

MTQSLNIRDLKEQTGCRVQPGLAGLYWYSVRFIEISGHFFYDYNVNYSFQRRILRLLYSTITFCVTLSHFLLSTIHIVLKTKDLRQVINAITTYAYSFLGVLKVLYLISRVKGFKRLLHYWDDVQPHPLFVNENENCEKTTYIRAKNIITFSITFGLVNCLFWIIKPFLINKVQEVNIGNDTTIIEEASLIVDSGYPFDRSSLATFISVYVFQVIWLFVCFMQITSIDVFLCCVIIHAIEQMNYLRKCAEKITDLSITLSHSGQTTGFSPEYTGYANTTRVPKYGRSINEENCQFKTSSNMYEKLPIAISDPDAKSKFWSAVTKSWADRHTKIIQFVDSIEETFGIVMCFHMFTATVTVSVLAYEASREETIGVQAGNLLGYIIHTLGQVFMFCYYGNELMEVSTNLGRDLYETPWDDATEDTHMFIKIVNVQCQKPLKLTGYKFFTLSYDFFTT

>TdomOr8J

MSKNSDFHKRPGLAGLYWYSIRILELGHHFSYRTKYTFCRSVIRVVYTCFSIIITYLHFVFTTVQIINTRTNFKEMVYTIAPATYAFHSVTKTSYLALRTKTLLNLLHSWDHVKVHPLFAADYEKFEKIAYSRAKKIVLLIFTLISINGIFWALKPLVSLRERKIDTGNETIVLKEKFLSVPSWYPLDPQENDANFYAAFLFQVFWMICAMLQVTSFDLVLCCMIIHAIHQMEFLSMIVKKIVALSASVKNAKKNEDMSLEKLPYDYRNDFLLLQQPPLRDDKQSTDSLNPNSQLDIPEDLDSASRTKLVSILIKEWVDHHRDIMRFVGKIEETYGFALMMHMALATMTLSILAYEASLEESLNMQVVNLLGYIMHTLGQVFLFCYYGDELIEVSNNLGPYIYETPWEEANNEVKIFIKVVNEQCKQPLKLTGYFFFTLSFDFFGTYVGAVVSYYLFLVQFK

>TdomOr9J

MLGFQSLQVCLQFNFRLLKVAGHWITEPAQDNNNRQNAALHWSYWLLLLYRVFITLITTIHVISVVAGCLKNADKFKDQPMIGAMGLFAFQALVKLVYIILKRAKIKNVLSTWNDTYTHSSFIWSRINAIESSTKSSKTVSTCLLASYVVLAIQWCLAPTSVSKDTTEEYNITTFINVTSSSKPLPFLAWFPLDFERSPIYTFIFAFQVVASLYFALIVAAFDGLFCALLSQAVNQMDHLRDSLGFLIDVCIENEPLLKDNIKSPNRTAIDNLASEIAGGLYRRYITSSSGSRIRPIELPYDQTTNKSTNLIVKSGRNKGFTHFGGDDYWENMRTSMSYCIHHHQYLIRFTDTLEELFSASMLIQFLYSTGLLCVLAFEATLIRGFDMKTFTLVVFLLVSVIQLFAICSYGNKILSESTRVTEEAYSKAWHKGSEDVRNVLQLIFQRSQRALVLSGANIFTVDLETFANVLAASFTYFMVLIQLG

>TdomOr10J

MVAKDNTVISFANFLAFHIKFMSISGHWLRLEPHNKWFTKKKVWFTLYRCFIRVFSDIHSITCFIEFLLNITDIEAATGNIVIVLYNTNAFVKMVYYSFRRRQFEELIENFNQEIEELHRLNDIRDAKRKTIATAHKHSKIIGSAIVTSSLLLVGKWCFFPLTVTQPPPDWKPDIAPNSTEEVVFRVLPANAWYPFDTLKTPVYEILAFFQTIGAVYSAFQLATYESHFVAILIYIVALMQHLRWSLGRLVEVSKVDEDIKTEDHSENTDNVRNSLYRDDPRVNVRANEIFTGPRSNNNSNKDVDDVLSYCVQQHQLILRFEKQINILFGPMVFMQFGISILTFCTLAFQATTIRGYGLKIMNLIEYLVVASVQLLTFSIFGNRLITESVSMVDDAYAKEWQKTTAKVKSTLKLIFLNSRKPLSMTGLGLYTLSLETFSSITTASFSYFMVLMQLR

>TdomOr11J

MQIKTDLRSFMRLTDRFMWYSGHFMNVSSTGKCFPLRMMLRAIYVLLARFFIDMHAITCTVEFLRNITDIDQATSVVVVIIFSFNTWFKTYYYTYRRNAFNEMFGLWSDTIDIPSSLREARASSIEAGRKHAKRISFLMMFSLVGMTGKWCLFPLTVAAPPSESIWSNGTLVTSEVRRPLPANAWFPFDPYVTPAYELVTTFQAVGAMYTAAQLASYDAFFVTLMIYAVAQMKHLKYSLGYLVDIDIQLSSQTKRNQKQTTQPAYLDNKRLNMSSFSEARGIDKTTGSEISVKSRDVQNRNENTMNNLEPSLEEHINVLDYCVRLHQLILEFCVELNRLFGVIVLEQFLVSTLTLCTLAFQASTITGYGLKVLNIVEYIICAVIQLLIMSTYGNRILVESVSLTNDVYAKDWHYCSPEVKSCLKLIFLNSKRRLQISGAGVYTLSLESFASIVTASFSYFMVLMQLRK

>TdomOr12J

MEDIKLHPMDHYTSAQIRLFKLLGLWPLDPSKVYGWRLVLHRLHTLYILSIAFLHSLCYLAKFLIYIHDIEQVAKNGVVMLYVLLGVMKQWYMLYKRRQIQSILRMWDNVMEDKRFVKDRVDVTTAMAAKCKKLTYSMWIFGLAMVVHWHLYPLFMNESAEQDIINNGNSSEVVRPYKYLPIEAVYPFDEQISPNYEIAYMLQNMMGPTILTTNVTFDNFFIVLLMLVSVQFDHVVNTLKSVSLRNSNSIFSNDRSRNRMIHSQLHGEDDGVDDSHDEFASGVVIKNEEDEAIYSLVMYCVKVHRESIRVANELEKLYSPFMLVFYLFFLIIMCLLAFDGSNMNDFGVKMFAMIEYLLLLVGELFVISYCGNELITKSTAVKNAVFESPWYGCSDKVKYAVRMMMIRSESAMKITGKGMFIVQMDAFSNVMGAAVSYYMVLMSFKE

>TdomOr13J

MLENTDERYFTDLEFCLNFILAFLRYVGLMPFTDETSSFIKKVGAYILMTLLSLPVIIQAITSSVQMFLSFEDLGKLATNAVLTLYVDIILIKNFYLYFNRERVLFLFQMWKNCRRSDVFDPLRLESIKKSTRFARNMWLLLWIPSFLMAAQWLFFPATVKTPPPDWDSANGTRIFRVLPASAWFPFDWSRSPAYELCYLQQAIVGILCVSQAGGFDSLFVSMIILNVAQLRHISECLSKFPSLVFPRQSTVSFHLNNDQGKRQMPRNSLEKIDSVGSFSLPYDGKEQSYKINISARGTYNLSHEDETKVTLAAKYIVHNHIMSIRFVEEVERFFTPVMLAQFVVTTLCLCLVTFEASTIQGYGSKIVSLVEYFAVTSLQMFILCYFGNQMITASSSVKEAAYASQWYSYGKKFKFCVRMIVLRAQIPLILTGGGIFTLSLETFKSIMAAAASYYLVLKQVKEDSSGLPIETPF

>TdomOr14J

MKISGENRKSFLTNLQLALDFNLKFLRYVGLIPHTNKTSSLVKKFLAYCVMILLALPITIQGIIGTVQLCYSFRDLGELATTAVVTFYIDTVFIKVYYMFFNRRKALRLFEMWNLCVKHEIFDDLRLESVEKSIRTSKIACLLLWLPPLFTGCQWLFLPLGQSAPTDWDSSNGTRPFRKLPAQAWYPFDYSISPAYEICYLQQAFSAFIPVCQAGAFDALFASMIIVNVAQFKHLSKCLSEFPSLVFPNSRQNNLKALPNTLLTVKDADRSTEQNPDLRDFVWRSKSIQNLPTEDEEKVASAAKYIIQNHALFLRFVEEVESFYSPIMLAQFVITTLSLCLVTFEASTIQEFSLKIITLVEYFTALSLQLFILCLFGDQMISASKSVKDAAYASTWYSYGKKFHYCVRMIVLRSQMPLRLTGGNIFTLSLETFNSIMTASVSYFLVLKQLREDSEGATTDN

>TdomOr15aJ

MYFTGNNFYTFVKSLFARKEVSAEVNNYVVKLLSAVGYWLEDLPCYSSSKISWFSFIYFLSSTSLLCLAFILNVLDLLLSEVPFILKTENIINALHLFKAMISAVVITLKRKELKSLPTVIINDNFVLFSHSENTPCCAYSMKLVIRIMSVMVFVVSLTGMSSWIYVSLNRTVCSAPSLVYNTSDNNCNTSAEPAVIRMWLPFDASASPAFEYVATFQIVCLCCYVLKNIGSTITIFIFLMKAGRSLEFLHRFLEILLSNGALGTLLRSSSSLVKNKSIINMASRPRSKFRYIANNNKLWNLSRSCISSQSVISTRLMNNNGINLWIKIHQNVIRFMESIESIFSLIIFVNVATDTGSLCLLAYTAANVHNQGGTAGAMLGFFFINTFEIFILCYLGSRIKNHHSSIISSIYRSAWMQNPVARQSDLQMIVLQCQQPLTISGAGFFTLSLELFLSIFGIVTSYFLVLVQL

>TdomOr15bJ

MFFIGNTFYTFLKSLFTKLKVNTKETNHVVNLLSVSGYWLENMPSYNSSKFSWFLFAYFLSTAFLVSCSLILNILALFLAKVQFVEKAEYIVNTLNLFKAMFTTVLITLKRKEIRSLSTMVIHDNFVRLPHTGKSSCLVYSMESAIRFIAVLAFVVPSGGMLFWIYTALNRDACPLSSLPYNISVNNCNNSFVPSIIMMWYPFDASSSPVYEYVAIFQIICLCCYALKIIGSDVTIFIFLFKTGRYLEILQKSMEELLSNRPLATSFRSTSTSIENNSVYNVTPRPRSKFGYVANKGKLWILSRSSVTSQSIIRKESINSSGLNLWITNHQNIIRFMESIESIFSLIIFVNVATDTGSLCLLAYTAANVHNQGGTAGAMLGFFFINTFEIFILCYLGSRIKNHHSSIISSIYRSAWMQNPVARQSDLQMIVLQCQQPLTISGAGFFTLSLELFLSIFGIVTSYFLVLVQL

>TdomOr15cJP

MYDTRNTFYTFLKSSFTKREVSSKVNNHVVKLLSATGYWLDDFPCCSSSILSWILFVYFVSTTILTCFSSIPTILGLFFSKVPLLQEADIIVHTINILKAMIATVVITLNRKEIRTLLTMIIKDNFVPLSHSGETCVYNIKSTNRFIASVMFGIPLFGMAFWIYVSLSQTVCPTSSLSYNMSVNSCIAVVEPFVLRMYPFDASVSPVYEYVATFQVACYYSYGMKIIGSDIILFISLFKIVRDLEFLQKSMEVLLSNISLSTTIFNLTPETRSKFGYNANKSKLQIFSTSIKVNSQSVISTQFVNSNGIHLWIKNHQKLIRFMESIESIFSLIIFVNVATDTGSLCLLAYTAANVHNQGGTAGAMLGFFFINTFEIFILCYLGSRIKNHHSSIISSIYRSAWMQNPVARQSDLQMIVLQCQQPLTISGAGFFTLSLELFLSIFGIVTSYFLVLVQL

>TdomOr16JC

MPFTKIVNINLVIVAFSTLFSPKCVFEDKASVLYWFRNTLVGILGSITVYHTSLSIIAIFMNIHDFTSVIFICMTLLYAFQALFRFFFVIINQKRIVKLMDVCSSLPQTCPEFNYSLKQAFECIKKESKKLFLMMFLGSEIVGLGYIGLPILRASILSNVTAHNEELPFKSGIAAKRPLITTCWYPYDVFVTPVYETEFFMEAIGTLWVIAVTSSCDSFLCSFIIYPIEMMEHLSRTILLITSDNLPTTHVRNNPFFSSSESISAIGYTMPSNCIRRGRNHDISQVNFIDIEEDIDNDQDSEGRSSAAKIIYQRRLLKAWIQQHQTITRTVDEIEAVFSPLILYTFLQGGIALCFLAYVAVRIRDLVPIVLITGYFFIIVLQLYTLSMHGERLMKVTSSPTNIMYDAPWWKCPRSIRQDIKMIYFRCQKPLKLTGMGYFTLSFEFFGS

>TdomOr17J

MQKMKLKEWRNSKMTSSGVIPFTSVAKPLIFMMLISAHWYPELTLRKKSIWYWIRAVYATVAISIPIYHVALATIDIFLNIKEFMNIIFVTMTLLYVVQGVFRVFFVILKKKSITKLVGMCSQMPAMCQDFEDSMTTASRNTGAKSLKLFLIMYIYIQMIAFAYIGLPFINAARLTNATMEENSPLKRGIAAPRPLIVTCWFPYDVFQTPVYETEVLLQAIGTLWTVGMVTTCDTLFCTFVIYVIEVIEHLGRMSIHITDETTRNTVHNCETFEKSESTINLSVSHKDIKGTFCENKGDTVAVKINPNIGKSNLIKWGQPYLTSKKKLLEVWVQQHQHILKTVDEIEAVFSPLILYTFLQGGIALCFLAYVAVRIRDLVPIVLITGYFFIIVLQLYTLSMHGERLMKVTSSPTNIMYDAPWWKCPRSIRQDIKMIYFRCQKPLKLTGMGYFTLSFEFFGSMLGAALSYFIVLMQLQ

>TdomOr18

MDVGEYTFEKLNKYTIRLLYISGLWFPDWPKQDPRWLMLPLRVLYFIFSAGFQSIHCVIGILDLMFQVSSFRDIIVNLVTTLYLFQAVFKTLFIFYNKGNLKRLVEMCNNVTTRQTKYITFQNSMAELHKGIEKDSKIMSHSIVDISLIVGSSFGLIPFVQSIFFQPTESDDMLYLNNTGGSTEFLTVSPLEIHHNESEIVTHPVARALVFRAYYPFDLAVDWIYGIVAFVQSYETVICVVVNTSCDALFFSFAVLIKHYLRHLQRISKMMRFASDFYGLPQDETIYNIKYQTHKVVVKNRKERSNTNKTNVTFTNDFFKLNDRSEGIMGYLQDSRNTRDEVVLWIVDHQTMLSLLDEINSIFAPLILFTSLQCMLTLCLLAYVAAMVDEAFFRFCISGYLIVVLTQTWTYSINGQGVINESVNLCKEIYGTGWYGYSDSEKNAMKIVCEVCKRELFFAGMGFFRLSTEFFTNTVGAAFTYFMVLIQFKD

>TdomOr19

MEQNKNKLEKLFSYNLKVFTVCGTSFPEWPVKNPKWWFYIIRIFYFMITAAIPLLQIPFTIMDLMYQVTVFQEAIMNMVVTLVLFLQLFRECFLFYNRKRMKRLFNAANILIENCGKVPKFRVSLEECLKETGKQSKIFTHLLVDILAIAGLLYAFLPLAQSFFFPSKFGNFSVDSNDSSIAAPTKRPLAFRAYYPVDLENEVIYGIIGFLQNMTGIIAVSIIGAGDSLFMSFVVFVRQLLRHLERMCQQIFLHPNTNIPLEEKPARSTMLPVDDSTTQEEIDVRVSGDDLNQQHNINGLTDKLSYEDSIKLRRDLIIWIQQHQSIISLYHEIEDIFSPLVLQTFLVTMMELCLSAYYAAMIDDYYDLLPLAGYQVAVFLQIFLYSFNGQRVINESENLERVVYESEWYCFNHVEEQAVKIIREICSKELSFTGMGYFHLTQVFFTNTVGAAVTYFMVLIQFKD

>TdomOr20J

MEKDKNKLEKLFAYTLKVAKICGNSFPEWQVKNPKWWFYVVRVMHFMMTAAVPMLHIPFTLVDLFYKVSSFQEVIINMVMTLVLIQTVVRELFYLYNKKRLKRLFDTSNILIENCGKFPGFRASLEESLSETAKHSSMFTHLLVDSLAIDAVLYISLPLATNIFFPTKVASFSVDSNETVDLVAVSGNRPLAFRAYYPFDLQSNWLYGIVAYFQYVSTIVSVSVLGDSDALFLFFSVFVRELLRHLQRISQQYFLYPNNKAFPRTKSQSHTRHPVDDSIFNKELDDRERGNILNNKDYENSENLSYEHSKNLRKELILWIQQHQSIISLYYDIEAVFSPLVLWAFLVCMMELCMSAYFAAVVDDYYDLLPEAGYLVAVLLEIFLYAIHGQRVINESVNLAKLIYESEWYSLNPVEEQAMKIIKEIFIKEVSFTGMEYFHPTQVFFTSIVTAAISYFLVLIQFKD

>TdomOr21

MGGDICSINKLFGYTIKLLKISGTLSRERRKESSKCIFYITRFVHYIITAGMPWLHACLGIVDLCLEVSTISTAIVNMQMSLFFLQATFRVLFMLYNTKAMKHLFEATDTLISCSKKDLKFQKSFETCLRITAKHSKIVTHFFVDFCSVIAVMYGFLPLSRSIFYPPASDDLSAQDNETERIVETTRPLPIRVYYPFDLQSNWIYGGVIFVQTWTIVMGVFVVGAGDALIFSFAILVRQLLCHLERLCVQLLIDTNNDKGSRITLCNAIAGDPDCLTPVEIRLCKKIKSQKTTQNNKNERSHTDLIEVSDCFKIELILWIEEHQRILSLFYEIEEIFQPVVLQIFVIAILELCLSAYLLATESDYFFLFCVTGYMFAALFQICLYAVHGQLVINQNARLVNIICEIDWTTFNLKEQQAVKLITKMCNKDLHFTGMSFFHLSLNFFTNTVAAAVTYFMVLIHFQD

>TdomOr22J

MEHDKPSIRKLFVYDVKLINLSGIIELPEEPIGRFKCKHFITRSVYFIITVGLLLLHAALAVLDLFLQVSAFQTAIENIITTLAFVQYVIRVGFLLSKKKQLVRLFEKTDKFFRKSSQHPEFQNSLEVCIEEIAKQSKKLTHMFVDTWIVTAFLYSLFTASATIIQLSIGNNIRHGEMNETDTVVHYERPLVFRAYYPFDLLSNWVYGIIVLFQTYVAVMVILSISSCDCLFLSYIIFIRQFVCHLERLCVQLFLATNLKNSALDFSMASRSNSPHLRKEVPDFYKRHETEIVRKNTIEDVGTILTNNHIKSSSGLRIELILWIKDHQRVLKLFYEVEDVFSPLVLQVFLIVISELCLTAYLIATVDEYFFLLSMGVYAFAILFQIWLYASIGQLVINESENLSRVFYKSNWYFFNAAEKEAVKLITNMSNRIIYFTGMGFFRLSQEFFTNVVSAAISYFMVLITFKE

>TdomOr23

MLHSEIEFMGKRFYEQIFGYNMKLLKLCGIINFAARPVDRVKCIPYITRTVHFIITVGIILLHTHLAILGLFLEVSSFQTAFENIIATLVYLQYMFHLCFILYRKKQLEILFNKSISFISNPSRYSEFQNSFEICIQETTSQTRKFTHMFVDTCIVFAFLYCLNSLSANIIFLPSIENNLFTELNETTAVVDDRKPPIFGAYYPFALQSNWVYGIVAIFQIYTTVLTVVTIASCDCLFINYTIFIRQLLRYLGRLYAQIGRENNPVNSRVTSAKVSSRVNLHAVPSEVALSKEVENNKTRIENTNFQNFTCINKIRDSAKLRTELAVWIQEHQSILSLFHEVETIFSPFVLHIFLLTIVQLCLAAYLVTRVGDKFILICLGSYLFIILLQVWLYAIFGQLVTNESVNLSKAIYENEWYTFNNSEKQVVKIISLISAKELFFTGMGFFQLTQEFFTNIVAAAISYFLILIQLKD

>TdomOr24a

MERENFPINKLFSYTIKLLNVCGILLVHFPQHQIGRHNWKFYIIRVSYSIFTLGLPLLHVLLSILDLFLQVSTFETAVENIMVTMGFFEFLFRISFMLYKQRQMKHLFKSTNTLIVSLSKRSEFQNSFESCFQKTIKESRKLTHVVVDSITILGFVYCFLPLSVNLFPESGNLSEGINVTECSIEVSRPLIFRAYYPFALQSNWVYIVVLLIQTYVTIMITVTVCTCDCLFFSYTIFIRHLLRHLERLCVQLLFDQKLKTFPGTQINVTTSDPLSPDQDTYFNKVIENGSINQDEITNDKIKQFLYYKEAQNSNERKITLISWIEEHRSVLRLFDEVEAVFSPFVLQIFLITIIELCLTAYLTTKVDNYFFFFCLGSYFGVVLFQIWLYAIFGQLVTNESVNLSRIIYESDWYNFNNSETQVLKVISRMAARDLSFSGMKFFRLSQVFFTNILTAAITYFMVLIQLKD

>TdomOr24bP

MEREKYSIEKLFDYVIKVFSISGLIFRQPHFDTLNTKHYIVLVGYSTITVGLIVVHALLSILDLFIQVSTFEEAVENIIITIIFLQYVFRVCFIIYKKQQLRILFEKANTLIYNSLRHRKFQNSFQICLQETAKQSKKLTHMFVDTCIIVALLYSILSVASEAIQFTSRDNSTSFERNETITIVCSRRQLVFRAYYPFDLQSNLIYIFVVIFQICIAVMMVLGIGSCDCLYLSCTVFIZQLLCHLLRLCELLFILNIHCKSEISSKEDVRNVPVNGILRKAMLCTELEYSNTTNMTYKPNSNEKECYSENTNEDSSEVRKYITIWIKEHQSILRLFDEVEAVFSPFVLQIFLITIIELCLTAYLTTKVDNYFFFFCLGSYFGVVLFQIWLYAIFGQLVTNESVNLSRIIYESDWYNFNNSETQVLKVISRMAARDLSFSGMKFFRLSQVFFTNILTAAITYFMVLIQLKD

>TdomOr25FJ

MEPFGDSFEDSSKYVTNLIRYSGHWVPYLRDPNTKIPKFVFVIITIFSFSMQAYHATTVTIRLIVTPQNFAKMIFNFAVFLYIWQVAFRVAVVAFKEKNLWNIFHRKTLQDVYNEDCRKSWKIVMTETHKKSVNMFIILTVFAIVAVSGKMYNVEETSDKKAAGDSDEKVANETETEEVPEITLIYDSWYPFEVNTPTRYWIVFGMQMYSLVHLAMLNFPVDAVYSSVLIRALEEIRHLQRMSDILGNTISKVIGDKQSSLTENMGDLELTDSGDVNRDVSAIVSSSQWDSMKFNVENRKKTNEPIPPEIMEILEQWILTHQSVQRLLADIEKTFSPVAFIILLVDEFVLCLIAYGAAKYDDKSAVTFFVAFMTFLIPELFIVCYYGQKICDESDLLPASIETIPWYNCPDSFKSTVNIILVRCKMLPKLTALGFFNVNVETFGNVLTVAVSYFMVLNSLS

>TdomOr26

MSANEDRALTLLWKIFPFIGFWFPSYRSSKSRRLYWFCYIYQVISLIICGWHTIMIPATSFIEYKALEPFLADLSLSLSVFQMGFRVFWISLKFGTFCTIYDLHRKIVNLRNEMSPKVQESWDIVHQESFRKAKVNFVLFGIPPILSTISRLLAPMADPKNDEDGVILNITAEADEGLRPLVANCIYPFDYSEDFAYAIAMVIQVFGCFTLVNSMVVTDLFQIFALIRLTEECHHLQRMADIMGSTVISVGNSKIGTPLAHKTTDPSSETTEDEVSAIVTRRKFVRDLKLSLDPDAYAMSQLDVDLRMWLTLHRLLIRFLEQLREITSPFILVTFLTNQCSLFLSAYTAVTITDPVFLTFHLCFNLFVFTQIGLPSYWGSKLTEEGVEISRSMYAVPWWNCSKSIQLTFKIILTILERPLTLTGMGFFTLSVEMFGSALGVAFSYFMVLRNLER

>TdomOr27aJ

MNSPCEDKILKFLKKVLPFLGFWTPTESRNFRTTGVHILLYIYYSVASFLMTWTSLGMIIGTLLDSDFNSILINSSLTIYATQNTIRYLLASYLSRTLPGIFKVFEEAPNVKNKFSSRIEESWNLLLTNSNKKTLKCVIIEFCSLFIAGGSRMILPMLLSSEDGSNSRKLSLNSWYPFDYKPSPVYELVQSVQMIYIIIAMMTICPTDLITYYLTVRFTEELKHLERISVLMREVTLKMGSESIVRGRRGQNESDISDQGRQGSTMNNSSHLDIDNTHETDVSQLLSFWIERHKLLIRLFDDIHRVVSPFIFFIFFTDQCVLFLVAYALVKVHDPVYVVFFFSFIVMVIAQLGIVSYLGNQVKKESRRTLRCLYNIEWWHCNKEIRQCIRIVQTRCKVPFMITGLGFFQLSVENFRKVLGVAMSYFLVLIQLEETGMV

>TdomOr27bJ

MNSPSEDKILKFLKKVLPFCGFWTPTESRNFSIMGIHILFYIYYFLFSLMMLWSWLSMVVGTVLDTDLNSILINSSLTFYATQNTCRYLHVSYIARRLPGIFKILEEIPNVKNKFSSSILESRNLLLSSCTRKTLKCVVIDLCSLFIAGGCRMILPMLLSSEDQGDTRKLSLQSWYPFDYQSSPVYELVQFSQMLYITFAMMIISPTDLITYYLTVRFTEELKHLERIAGLMRGITLEMGSESIVHCRAVKNVSDISFQDHEEKAMDISSISDLNNTHEADVSQLLSFWIEHHKLLDRLFDDIHRVVSPFIFFIFFTDQCVLFLVAYALVKVHDPVYVVFFFSFIVMVIAQLGIVSYLGNQVKKESRRTLRCLYNIEWWHCNKEIRQCIRIVQTRCKVPFMITGLGFFQLSVENFRKVLGVAMSYFLVLIQLEETGMV

>TdomOr27cJ

MDSPCEDKILKFLKIILPFCGFWTPSNMRKYSVGGIHIFFSIYYTVASFFMIWSWVSMVIAAFFEFDFISILTSASLVIYGIQNTMRYLLVSYKSRKLPEIFRTLGETPNVKNKFSSTIRESRYLLFKSCTRKTVRFITIIFCSLSIAAGTRMILPIFISSEDQSGSRKLSLNSWYPFDYQPSPIYELVQCSQLIYILFAMATILPTDLIIYYLLIRFTEELKHLERLSKLMREVTLAMDFTSDFSSLKGEGQLVINNDCREETIIDSPDLVINNSHQEDDFSDLLMCWTKHHLILVRLFDDIHRVVSPFIFFIFFTDQCVLFLVAYALVKVHDPVYVVFFFSFIVMVIAQLGIVSYLGNQVKKESRRTLRCLYNIEWWHCNKEIRQCIRIVQTRCKVPFMITGLGFFQLSVENFRKVLGVAMSYFLVLIQLEETGMV

>TdomOr27dJ

MLNLLRKCIFLFGVFTATNVKKKSQVLSLLLYSYKFTISVLMFGHTIGTILRSMLETHFETLLINLSLTIYITQILLRYIIVNASSERSLNILNRFKTTSNFSNMLSSDVKLSYDRMIARCNRNILSITIILMTAYIPTVLSRFISPSLATTDSSENKTVGSDLSQSRKLLIECWYPFDYHPSPMYEITLCFQTVVSLFATFIVITTDLIINYLLVRLIEEYKHVQRLLPMLIKMADIGQELTEKKVLFQKDDIISIISIDSTDDTDTIYRNKISGQCAEDVIFGEHVKIWIKYHLSLTRLFDDIHRVVSPFIFFIFFTDQCVLFLVAYALVKVHDPVYVVFFFSFIVMVIAQLGIVSYLGNQVKKESRRTLRCLYNIEWWHCNKEIRQCIRIVQTRCKVPFMITGLGFFQLSVENFRKVLGVAMSYFLVLIQLEETGMV

>TdomOr28J

MDFGLEAALRPIMILLHLSGHWLITWPNHSRDWPKILKVIHSVLVTIMMTLLVLGNIVDFLSTIYDIRCLVVNGLMVAFFVQGFVYLISIMLHRTSFLKLINLCKEFENCLFFRKSRRCVRKRMVFRSRKTLIVSSILSCTCASLWVYPLLTYKPLVVNGVRYQNSNFSNFSWVFHLSYWYPLENVSSPLYELVLIFEVGSLYVAFLVVLACENLFVTLVLYPLEEVRHMRKIFQAVLNFCDQHLISYNYAEDNDEFSDNIETFKQCKNKSLHTRGDKTRSTMHVSMPQDRNASEYKECWKILFPQLINSNVNNEEDITSEIERDAWKLVESYPNGVEEAVKKCIIHHQTIIRLTSAVEARLADILVTVILSSGCILCMMSYIVFATAKAYPNLVLALLISFIVTFHRTYLACRYGTELIEESREIFFTAYNSPWYEATGNVRFYLEIICQRTQTPLSITGGKFFTVSLGLFGKIMGVILTYFVVLVQLNYNNTTSCVNNQNETDNKT

>TdomOr29J

MSRMSNANQLIGRILSPLLFMCELSGYWISWTHDNRKYTGSYSKRIIKNTLKLIYFIIVNCFGVAIALAAVADLWTKIEHFQEAVYNLISTIYVLLSAVRSLFFALKRRQIIKLISYCNTKVPSCILKDLEREKYRRFACRFKNISIIYSTITILSICTWNVVPFVTSSIEIKEENGRNVSHIRIMGLSTWYPFDTRYSPSYELSIGFQLLGSILINLRCVTCDILWLGIFLFIAEELSVLSYCIENTMLSAYYGVKKNFQAWSSVGALEYSHNRPNADRRYCDKLHNIPSNILLYRDVCPKFGSPPNALENILPEWIILHQNLRRYVYEVNETISPFLLTMFLFSSVNLCLQAYLASRVRGNIAMAISVITYGVLLLLQMYLYCKYGSDIRQRSLGFGSTLYDTPWYQHSSSEVSYYIQVIMLTTQTPMSLNGAGFFSLSLELYASILGAIFTYFLVLLQLT

>TdomOr30FJ

MVSCVDKTFQIPIFTGAISGFTLIRDPNHMTTVRCKNALDLAFNIFMFLATLLLLIFGCIDLVFKVSDISLVLFNLLTTLYVLHTMVVRVFGILKRTQVSKIVNEFKLLTDISVSKDFQSNHTENLCRQFRTFVYTYISVFLIASAYWTTLPFLVRDEQSGNSTSGVITQVMVSWYPFDYTTTPGFEISIAFQVFILFQNSFCIFSCDGTVFGCFMLLLSELKRLSFTLRQLIASNDSRVKWKNRLNRINYPLSYQNESSVYDVIRASLAADREVNDFIVINKPWKKEELKRKLTVWLQIHQKLYKLSTEINRNISVLLFAVFFIDGLTICLLAYMATQVNNVIGATSIVLYLLVLLAETYLYCAYGNQIKEKSMDLAKALYDTPWIYVASDEIPVLQIISQRCQMPIIFTAGGFFPLTMDFFAGAIGTIFAYFVVLLQMPVNKK

>TdomOr31aJ

MSGTTFLRENSWAIQNMKGFFHVGRFVGLWVPSQELEKNIFKKYAGYIYNFIMCINTFVLLTFAAIDWSLKSPAFVPVAYNSLSSLIIIQTFVKIVHSLFRRRFLFEVLKKCDYISNTSTFFVPLQKLSFEKGERFYRNTFFILPITFVACGSFWCSLPFILKNSSELSHNETYYRSQILASWYPFDYSTSPGYEISVAYQIIEALSCSVIIVVSDGYFIAFYTYIVAQSHFLGESLKQILVEFDSGIFRGVQRRGPTLKHRRFLTLVVESSVVPDNQQIYSSGFDCTTALTLKETRRYLHEWISYHQSTIRLCDEINSITSPVVVLIFLVDGLILCLLAFMATRVNDVVSVCSLLVYFVLILAQVYLYCLCGDKVKTQSESLADVVYNARWMDLDPDTIQILQMISQRCQKPLTINGGSFFTLSREFFLAMMGSVLTYFIVLTQLPDRQDHENK

>TdomOr31bJ

MKILNLEDLFKFPAYLGCATGIYNPNKLSKYECFRKFNKVYCVCMIFDSVIFFISCMIDLSLKTDDHIQLIFNILSTFHQALTSFKYIYFYVRNSKLQQIIKRSNKMVNGLFYHRMNKWNLRNIHFRFQIVMFIYFLVGLASYCSWIATAFYQAAQQNSTETSREVQVRTSWYPFDRISSPGYEISISYQIFESFKGVFSTICTDVCFFALFSYLSSQVRFLQAAMKEVLRTIGMQKCADNKRCKRPISNRKLSDPVIYVRSEQITKEEIQTNIHMRFDLKQGLGHWLDVHQSVIRLCDEINSITSPVVVLIFLVDGLILCLLAFMATRVNDVVSVCSLLVYFVLILAQVYLYCLCGDKVKTQSESLADVVYNARWMDLDPDTIQILQMISQRCQKPLTINGGSFFTLSREFFLAMMGSVLTYFIVLTQLPDRQDHENK

>TdomOr31cJ

MYIENLLKFPAFLASFAGVYIPCGKYWKHDTNICNYLRSIYVTFTICDLFVFSILSIIDLGLKVDEYLAFTFNLLSTFYSLQVLIKTVYFSLKKDKTKLVARIWNKLVNGTFFYALQTRCSKRLRHRYHIILILYTLLAFSSSVLWVVVPFVIDYKQNSTATLYRGQVKNSWYPFDIMSTPGYEISISYQIDQCFKSFLITSSTDMCFFLYFLCVQSEIEFLEAAIKEILPSTGTDILVYDKYKSLPRSDIPFCVRRPDLRNRTSECNCVCGLHQGNIVQSLIWSQSQKKLHHWLTLHQTLIRLCDEINSITSPVVVLIFLVDGLILCLLAFMATRVNDVVSVCSLLVYFVLILAQVYLYCLCGDKVKTQSESLADVVYNARWMDLDPDTIQILQMISQRCQKPLTINGGSFFTLSREFFLAMMGSVLTYFIVLTQLPDRQDHENK

>TdomOr31dJ

MSEHIQIKKKPWIVHNLLKCFTYLSPGVGLWIPWQLQEQCSVQNKVCSFFNCIMGLDTGIAFILSLVNFILKLPAFLPAVYSSLASLILLQCLGKTVYVFVRRKRIFGLIQNCDHISIHSSPLARFEGDSFSKLERCFRSTSCIFACYYFISASLWIVLGFTKMKVDKANEDIYCNQIFQSWYPFDYQSSPGYEISIAYQIFEVLKISSLLIISSMYFVAFCICLEAQLEFLEESMIKILEELNLNLSLNEKAGMCHRELLPVLMQNDVFCGNQHACCRKSKARPSSTLEETRAYLREWLSFHQSAIRLCDEINSITSPVVVLIFLVDGLILCLLAFMATRVNDVVSVCSLLVYFVLILAQVYLYCLCGDKVKTQSESLADVVYNARWMDLDPDTIQILQMISQRCQKPLTINGGSFFTLSREFFLAMMGSVLTYFIVLTQLPDRQDHENK

>TdomOr31eJ

MHTWSMNSILRSVKHIGIFAGLHIPCDLTEQSKVCSNLQKCYSIFHICDSGIFLLLSIIDLFLKFNNILDLIFSIISTLHVIQTLLKPLYFLLLTKAFRKVFMKLNQIANQSSLELVQKQVLLALSNKCFIVIYVYFLFHLLSLFGWIVAPFYGASEKSSMEKFRCHQVRSSWYPFDYSSSPGYEISVSYQIFQSIKGSLVRCSTDACMFVLVLFVESQLSLMRDALAEILRSNELEQCRSDSKCSSPVEDRHDSLHCPTCGCNQRYWIQSNEAKSLKSKQFTKLREWLEAHQSLIRLCDEINSITSPVVVLIFLVDGLILCLLAFMATRVNDVVSVCSLLVYFVLILAQVYLYCLCGDKVKTQSESLADVVYNARWMDLDPDTIQILQMISQRCQKPLTINGGSFFTLSREFFLAMMGSVLTYFIVLTQLPDRQDHENK

>TdomOr31fJ

MPDDICGKNKPWIIQNLLICFIHLARIVGLTLPCQEPRMSVLKVCLQRVFSIIMCIDNGIIFVLAIVDWSLKLPAFLAVIYNSLSTLFILQAFIKMVYLSFRKKLLSDIFKKCDSVSNNSTFFALLHKQSLNSGERCFKKTFYFLTAIYFLSATFWSALPFAVNSSGELSQNETVYRSQVMASWYPFDYNTSPGYELSMSYQIFQTFKCASIVLISDGYFIAFYVYLAAQANFLGRAMNKILLEFQSNIFRCVKRRGSKLKHRRLLSIVVENSIFPDNQELCSCESDCSGALTLNEREKYLREWLEFHQSTIRLCDEINSITSPVVVLIFLVDGLILCLLAFMATRVNDVVSVCSLLVYFVLILAQVYLYCLCGDKVKTQSESLADVVYNARWMDLDPDTIQILQMISQRCQKPLTINGGSFFTLSREFFLAMMGSVLTYFIVLTQLPDRQDHENK

**5 *Machilis hrabei* ORs**

>MhraOr1F

MREKTVFETVGKKWRVRNKVKQLRQNNEFQDVQNFTRRIGTLMVASGYPLFMFSNSVRCRFKCKLHIFYSGAIISIVALHLLSCCVDLCFSKNASEAIFNILTTSYILQCFLYYVFVIIKRRHFENLIDCIFVKEIPTIYVESNTFQAKSVLERSKVRIFALVMLSLFGWFVWSIFPFVITLVKPYSLASTNDNITIPLRISASWYPFDITTSPVKEIIATLEATILLWSMSAITSIDMLFCISMVTLVEYLKCLGRNLKFLSQHSVHRISQTLNQTRHTTCSPIKSSTEHAIQFTYLESLKMKNLASVNSKDTENEPGKRSTESIHSFHCKHDSTLEQLGLWIDSQVHICSIVQEVQSAYSFIFVNAFLLNGLEVCIITYILAAVSMPTSRVVGMVLYMVCVLFRTFLLCHLGTELTDQGLNVCCAGHSSTWLNAPDQIRSTLQIILTRSQMPLSITGAGLFTVNLPFLASMVSAIVTYFIVLLQVNKSS

>MhraOr2FJ

MTICDVISRLKRTLILVAIAMFKKANFRTSVKQLQKKDDIQKLQTVRKRVWTLMRISGHFLFVFPQNEVTRSKSYGQIFYSLTAISLTALQLACCCVDLCFSRTTSAIIFNITTSSFILQSFLYYIFVVTNRSKFVKIATLLDENKIPKALVGLKSIELKPALRHSKVHVQALVMVSAFGWFTWAVFPSLVDWISPESGVVRSFCNTSLVSIRISPAWYPFEVCKTPMKEITTVVEGLILLWSIWAVASVDMFYCTAMATAAEYLKCLRKTLKSISRHHRTTSQEDCSCSPQNTRHNDGHKFAFISPDELNPLTNIDEDSMEKGSREDASEMFPSTQPLCNSVVEDLRLWIDCHVHACRIVQEVQSAYSFIFVNAFLLNGLEVCIITYVLAAISLPNVRIVGFVFYLVCVLSRTYLVCHLGTEVTDQGLKVCCAGYCSTWLSASDQVRSALQIILTRSQMPLSITGAGLFTVNLPFLASMVGVIVTYFIVLMQVHHST

>MhraOr3F

MFYIFFQSWSNPHQDYYLHTINKTMNEIFGKANVVYLEYTTNNRVLRKWVERLVKLMKISGHWPLRMPGEIRSPLQNKFQVIYCSIVITLALTYCICYCTALFTSRSSQLALNNISITSFIIQCCICYIVFVVHKRKLETLLNYIFENDMPECPRRYKALSVKMLLSRCKFLILSLGIIDSIGWLIWTLFPFAVSSANSNRTSLRFAEAWYPFDSMTSPVNEIISVYEAVIMLLIITATTTCDMLFCVVMTFVSEHLKCIGKALDCRVDGISHDVSRHHYLQAEKLLVSELERKVVTIDTGLIHVPIMLHTDKGNYKCHQLSASQIVTRCDSVERLGATIDSHVKIFRILETVQSVYSFYLLTLFLTIGLALCIMAYILASADNSLTRIAGMVCYMTCAFVRIFLLCLAATDVTEQGQNLSRAGYSSKLALVSDGVRSTIQATATRAHIPLCITGGRFFTVNLSFLASMTSVIFTYFIVLLQVNGKQH

>MhraOr4FJ

MNYNYRSTKVASFNKTNSNTDIQKWIKRMRILMSISGLWPLSLPNENEGTFQKIRHIIYSCCILTLGCMFCVTYCIALYLSRNAQHVLNNIIMTSYVFQGCVCYIVRIVKRKNFEALLNYISENSIPECPKTHRFVASKTILTRCKSTILSLGIVTLPAWLGWTLLPLILLAVDPGVSSSNHTELRFAEAWYPFDAKVSPTNEIITIYESILLLLFIWSIASSDMMFCLVMTLVVEHLKCLGKNVECVVDGITQHAPKLQYLGEERFPKESNKLDNFVVRKHVSGPSTHFHPSRDRSFTLKCSNSSDISRNRVEFESLTSTVDSHVKVCRIMEVVQSTYSSYFTIMFFSSGLGFCVMTYFLAVGTNSTSRIVAMIIYMAAVFRRIFFLCVLATDVAEQGLNLCKAGYSSKLISAPDHIRSALKILTSQAQIPLCITGARFFTVNLTFFVSFAGVIFTYFILLIQLNGRQ

>MhraOr5FJ

MKIDVDSVDHTDDYIHLRKWIKRIGIILRISGHWPFRLPHEKRNQHKSKFRQVYSCLVITLGFITCSCYCIGLCLSESIAQALNNITVTSYFLQSCVCYVSFIINSRKLETLFNYLFENEVVGCPRGYKMSSIKTTLFRCKFVAFSLGILSFFGWLMWTLLPLAVLVVDSGATGGGNQTSLRFVEAWYPFDTTTSPMNEVIAIYEAVAMIFLITAPMSSDIMFCVLMIFIVEHLKCLGMAIECTLKGISTNQHQNIGFDDSVSDVNVQRRIVIGKESPIQSIHVPIKECSRQSSDAVFREKRHGTHHQIIRSHNYTDATSLCNIVDSHVKIYRTMEIVQSVYSSYFATLFFTSCLAVCALAYFLAATSTSFTRVPGMVLYLMYIFLRIFLLCLLATEVAEQGLNLCHAGYSSKLVLASDHVRSTIQAIATRAQIPLSITGARFFTVNLSFLASMAGVMLTYFIVLLQVNAKPKP
