# Supplementary figures and images for: The origin of the odorant receptor gene family in insects
Source: eLife. 2018 Jul 31;7:e38340. doi: 10.7554/eLife.38340 (PMC6080948; doi:10.7554/eLife.38340)

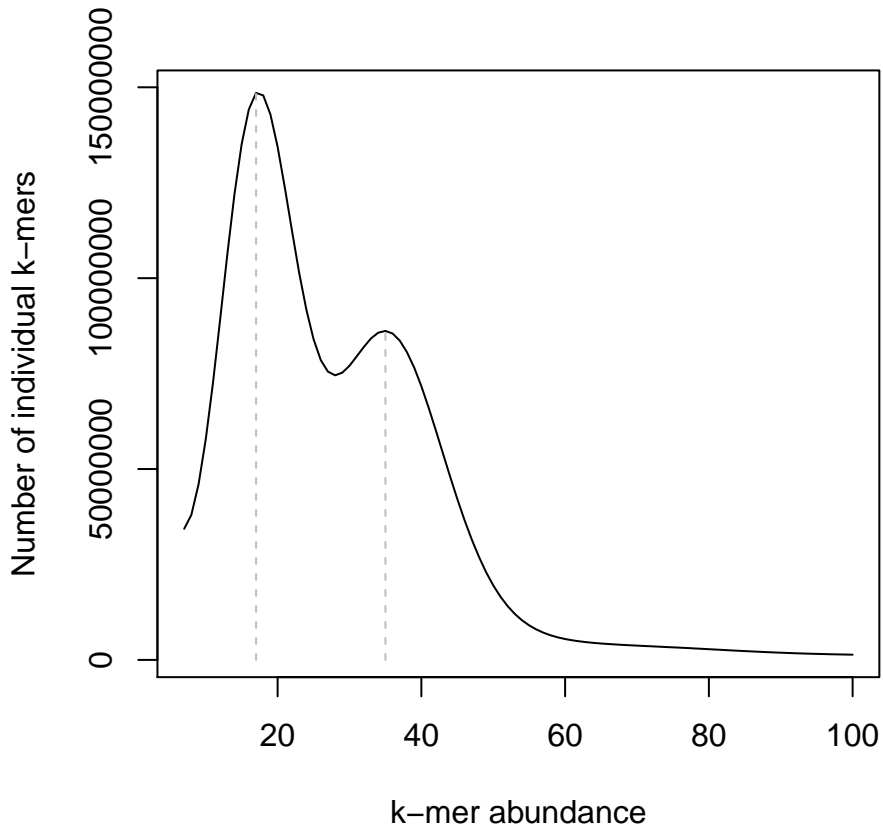

Supplement: Supplementary file 2. — A high heterozygous peak with a maximum at k = 17 in comparison to the homozygous peak around k = 37 indicates high heterozygosity in the genomic data. High heterozygosity is a known culprit to difficult genome assembly but cannot be avoided in most non-model organisms, which often cannot be used to produce inbred lines. [file elife-38340-supp2.pdf]

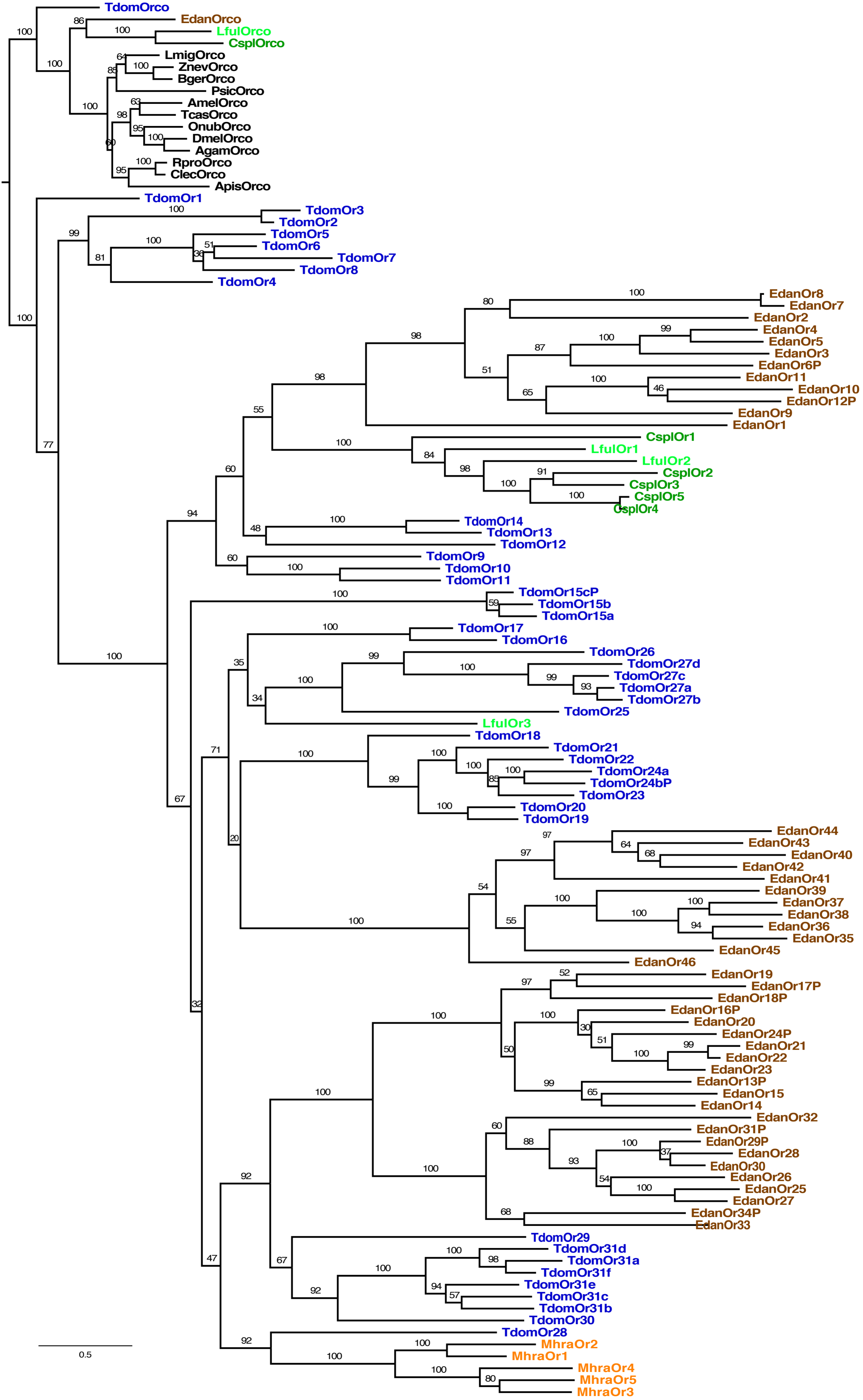

Supplement: Supplementary file 3 — The Maximum Likelihood phylogeny shows relationships between the ORs and Orcos detected in M. hrabei (orange), T. domestica (blue), L. fulva (bright green), C. splendens (dark green; Ioannidis et al., 2017), and E. danica (brown). [file elife-38340-supp3.pdf]
